# Effects of saffron (*Crocus sativus L.*) supplementation on cardiometabolic Indices in diabetic and prediabetic overweight patients: a systematic review and meta-analysis of RCTs

**DOI:** 10.1186/s13098-024-01530-6

**Published:** 2024-11-27

**Authors:** Mehdi Karim, Samira Pirzad, Niyousha Shirsalimi, Mohammad Hossein Hosseini, Pouya Ebrahimi, Sara khoshdooz, Pegah Rashidian

**Affiliations:** 1 Faculty of Medicine, Bogomolets National Medical University (NMU), Kyiv, Ukraine; 2 Faculty of Medicine, Islamic Azad University, Tehran Medical Sciences Branch (IAUTMU), Tehran, Iran; 3 Faculty of Medicine, Hamadan University of Medical Science (UMSHA), Hamadan, Iran; 4 School of Medicine, Shahid Beheshti University of Medical Sciences (SBUMS), Tehran, Iran; 5 Tehran Heart Center, Cardiovascular Disease Research Institute, Tehran University of Medical Sciences (TUMS), Tehran, Iran; 6 School of Medicine, Guilan University of Medical Sciences (GUMS), Rasht, Iran; 7 Reproductive Health Research Center, School of Medicine, Guilan University of Medical Sciences (GUMS), Rasht, Iran

## Abstract

**Background:**

The incidence of diabetes mellitus (DM) is increasing worldwide, and there is growing interest in the potential use of natural compounds as an alternative treatment for managing DM. Several research studies have investigated the impact of saffron consumption on managing and improving metabolic profiles in patients with DM, and they have shown promising results.

**Object:**

The study aims to systematically review and perform a meta-analysis to evaluate the potential effects of saffron and its extract on cardiometabolic indicators in diabetic and prediabetic overweight patients.

**Methods:**

We conducted a comprehensive systematic review and meta-analysis, searching PubMed, Scopus, Web of Science, Embase, and Google Scholar for all relevant studies published before April 20, 2024. We extracted weighted (WMD) or standardized (SMD) mean differences (before-after) and 95% confidence intervals (95%CI) of the outcomes and conducted meta-analyses using R. The study protocol was registered in PROSPERO (CRD42024538380).

**Results:**

Out of the studies screened, 15 RCTs were selected for inclusion in the systematic review and meta-analysis. These studies collectively involved 869 participants, 438 in the intervention group and 431 in the control group. Notably, our results showed that saffron supplementation led to significant changes in FBS (MD: − 8.75 mg/dL, 95% CI [− 14.75; − 2.76], P < 0.01), HbA1C (MD: − 0.34%, 95% CI [− 0.39; − 0.9], P < 0.01), TG (MD: − 13.28 mg/dL, 95% CI [− 22.82; − 3.75], P < 0.01), SBP (MD: − 5.33 mmHg, 95% CI [− 8.99–1.67], P = 0.04), DBP (MD: − 1.02 mmHg, 95% CI [− 3.91; 1.86], P = 0.03), AST (MD: − 1.32 IU/L, 95% CI [− 1.72, − 0.93], P < 0.01) levels in T2DM patients compared to placebo or no supplementation, indicating its potential as a therapeutic intervention. However, there was no significant effect on Insulin secretion (MD: − 0.15 µU/ml, 95% CI [− 2.1763; 1.8689], P = 0.88), HOMA (MD: − 0.35%, 95% CI [− 1.34;0.63], P = 0.48), TC (MD: − 4.86 mg/dL, 95% CI [− 9.81–0.09], P = 0.54), HDL (MD: 0.18 mg/dL, 95% CI [− 0.93; 1.29], P = 0.74), LDL (MD: − 1.77 mg/dL, 95% CI [− 5.99–2.45], P = 0.41), TNF-α (MD: − 0.34 pg/mL, 95% CI [− 0.99–0.30], P = 0.29), creatinine (MD: 2.83 mg/dL, 95% CI [2.29, 3.37], P = 0.31) and BUN (MD: − 0.44 mg/dL, 95% CI [− 1.43, 0.55], P = 0.38).

**Conclusion:**

Saffron may improve specific CMI indices in overweight patients with diabetes or prediabetes, including significant reductions in FBS, HbA1C, TG, SBP, and AST. However, it did not significantly affect HDL, TC, LDL, insulin secretion, HOMA, DBP, TNF-α, ALT, Cr, or BUN. Further research with more trials and extended follow-up periods is needed to confirm and expand these findings.

**Supplementary Information:**

The online version contains supplementary material available at 10.1186/s13098-024-01530-6.

## Introduction

Diabetes mellitus (DM) is a prevalent metabolic disease that poses a major public health challenge and has a significant impact on human well-being and healthcare costs [1, 2]. DM is estimated to affect approximately 530 million adults worldwide, representing a global prevalence rate of 10.5 percent among adults aged 20 to 79 years [3, 4]. According to the Centers for Disease Control and Prevention's DM Surveillance System, around 37.3 million (11.3%) of adults were diagnosed with DM in national databases in 2022 [5]. DM alone is responsible for more than 1 million deaths annually, making it the ninth leading cause of death [6].

The utilization of herbal medicine for various diseases has a long historical background across different regions worldwide [7]. Herbal supplements are increasingly investigated as alternative treatments, with growing literature supporting their use in diabetes management. Various medications and herbs can manage glycemic indices and other metabolic markers in prediabetic and type 2 diabetic patients. For example, a study on okra reported benefits on fasting blood sugar (FBS) but no effect on glycated hemoglobin (HbA1c) [8], highlighting the potential limitations of okra in this patient group and the need to explore other herbs, such as saffron, which may offer broader benefits.

In recent years, there has been growing interest in the potential therapeutic effects of natural compounds, particularly herbal remedies, in the management and improving patient outcomes of DM [9–12]. Among these natural remedies, saffron (*Crocus sativus L.*) has attracted significant interest as a natural product due to its promising effects on glycemic control. Saffron is rich in bioactive compounds such as crocin, crocetin, and safranal, which have antioxidant, anti-inflammatory, and blood glucose-lowering properties, which could potentially contribute to the therapeutic effects of saffron in the treatment of DM [13]. In vitro and in vivo clinical trials and studies have revealed saffron's antidiabetic, hypolipidemic, and anti-hypertensive properties and constituents [14–16].

Multiple clinical trials have been carried out to explore the potential benefits of saffron supplementation in the management of type 2 diabetes mellitus (T2DM). These trials have indicated that saffron supplementation has the potential to improve glycemic control by alleviating T2DM symptoms [17]. Additionally, saffron supplementation has effectively regulated FBS and HbA1c levels in patients with T2DM [18]. Additionally, saffron supplementation has been associated with beneficial effects on lipid profile parameters, including total cholesterol (TC), triglycerides (TG), low-density lipoprotein (LDL), and high-density lipoprotein (HDL), which are important risk factors for cardiovascular disease in diabetic individuals [13, 19].

While recent randomized controlled trials (RCTs) have explored the impact of saffron on cardiometabolic indices in individuals with diabetes and prediabetes, the literature in this domain is characterized by variations in sample sizes and inconsistent results, including positive, negative, and non-significant findings. However, a significant gap remains. Addressing this gap could provide more definitive conclusions and practical recommendations for clinical practice. This prompted us to undertake a comprehensive systematic review and meta-analysis involving gathering data to address these discrepancies effectively. This study aims to review these studies and delve into the potential impact of saffron consumption on the cardiometabolic indicators in diabetic and prediabetic overweight patients.

## Materials and methods

### Protocol and registration

This systematic review and meta-analysis were performed according to the Preferred Reporting Items for Systematic Reviews and Meta-Analyses (PRISMA) guidelines [20]. The protocol of this study was registered in PROSPERO (CRD42024538380).

### Literature search strategy

A comprehensive systematic advanced search was done in scientific electronic databases PubMed, Web of Science, Scopus, Embase, and Google Scholar from inception until April 01, 2024, to find studies evaluating the effects of saffron and saffron extract supplementation on cardiometabolic indices, including glycemic markers, lipid profile, blood pressure, liver enzymes, inflammatory markers, anthropometric indices, etc. in diabetic and prediabetic overweight patients. Relevant keywords from Medical Subject Headings (MeSH) and non-MeSH phrases were used to search database sources. The search technique was based on "Title & Abstract" and Boolean search words (AND & OR). No time and no Language restrictions were applied to the search strategy. In addition, we performed a manual search of all relevant article reference lists to ensure the identification of potentially relevant trials.

The exact query words for saffron and cardiometabolic indices are presented in supplementary materials.

### Selection and exclusion criteria

The PICO criteria (Participant, Intervention, Comparison/Control, Outcome) were used to search for items related to saffron supplementation, and diabetic and pre-diabetic patents used for the present updated meta-analysis are presented in Table 1. In addition, time and language restrictions were not included in the study.

**Table 1:** The population, intervention, comparison, outcome, study design (PICOS) criteria

Domain	Criteria Selection
Participants	Diabetic and pre-diabetic overweight patients
Intervention	Saffron and saffron extract (*Crocin*) supplementation
Comparison	Placebo/Control, no treatment
Outcome	Clinical and Seum changes in Cardiometabolic Indicators (including FBS, HbA1C, insulin level, HOMA-IR, TG, TC, LDL, HDL, SBP, DBP, AST, ALT, ALP, Cr, BUN, TNF-α, BP, BMI)

Non-randomized studies were excluded, including cross-sectional, case series, case studies, case–control, and cohort studies, review systematic and meta-analysis, abstracts, letters to the editor, in vitro and animal studies, and studies with less than one week of follow-up after intervention. Also, studies on non-diabetic and non-prediabetic patients and other parameters were excluded.

### Synthesis methods

The results are different since recent RCTs focused on the effect of saffron and crocin (one of the active ingredients of saffron) on blood markers. They cannot provide a basis for clinicians to formulate treatment plans. The present study decided to include crocin and saffron trials. Subgroup analyses were performed for age, gender, BMI, dose of supplements, and duration of intervention. Subgroup analysis was performed to evaluate the impact of each on glycemic markers and lipid profile alone.

### Data extraction

The authors agreed on the inter-rater reliability of the data screening and selection process using a kappa statistic. Three reviewers (SP, NS, and PR) independently extracted the data from the included studies in a predefined Excel sheet. The extracted data included the first author’s name, year of publication, study location, study design, sample size, participants’ mean age, gender, body mass index (BMI), form and dose of saffron and crocin, duration of intervention and mean ± standard deviation (SD) (or mean ± standard error [SE]) of change in serum cardiometabolic biomarkers (FBG, HbA1c, Insulin level, TG, TC, LDL, HDL, BUN, CR, AST, ALT, TNF-α, etc.) in each group of intervention and control.

### Quality and risk of *bias* (RoB) assessment

**Fig. 1:**
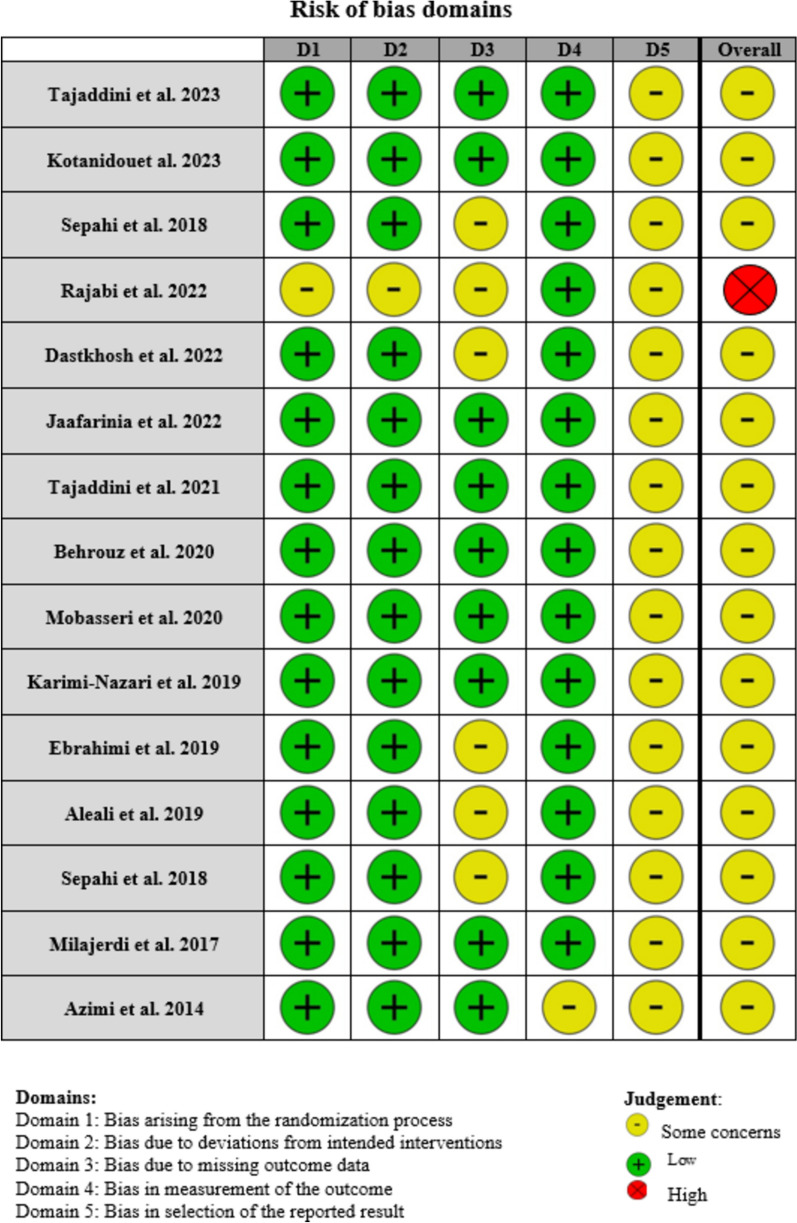
Quality assessment (A summary of the risk of bias according to Cochrane criteria)

The quality of the studies was evaluated independently by two reviewers (M.K. and S.P.) using the risk of bias according to Cochrane criteria. The risk of bias assessment version 2 (RoB-2) [21]. The included studies were conducted using established criteria to ensure the validity and reliability of the findings. Key domains evaluated included selection bias, performance bias, detection bias, attrition bias, and reporting bias. Each study was meticulously reviewed for randomization methods, blinding of participants and personnel, completeness of outcome data, and selective reporting. Studies with unclear or high risk of bias in one or more domains were noted, and their potential impact on the overall findings was considered in the analysis. This comprehensive assessment ensured the systematic review and meta-analysis conclusions were based on robust and reliable evidence (Fig. 1).

### Statistical analysis

All data analyses were performed using R software (version 4.3.3) [22], utilizing the ‘metafor’ package [23]. Effect sizes for each study were calculated using weighted mean differences (WMD), presented with 95% confidence intervals (CI), employing a restricted maximum likelihood (REML) approach within a random-effects model to account for the observed heterogeneity. The inverse variance method weighted each study’s effect size based on precision. Cochrane’s Q and I^2^ tests were applied to assess heterogeneity among the included studies. A P-value of 0.05 or lower, or an I^2^ index of 50% or higher, indicated significant statistical heterogeneity. Publication bias was evaluated through visual inspection of funnel plot asymmetry and Egger’s test. The trim and fill method was used to adjust for publication bias indicators. A meta-regression analysis was conducted to investigate the effects of study duration and dosage on the outcomes. A P-value greater than 0.05 was considered statistically significant.

## Results

### Literature search and study selection

**Fig. 2:**
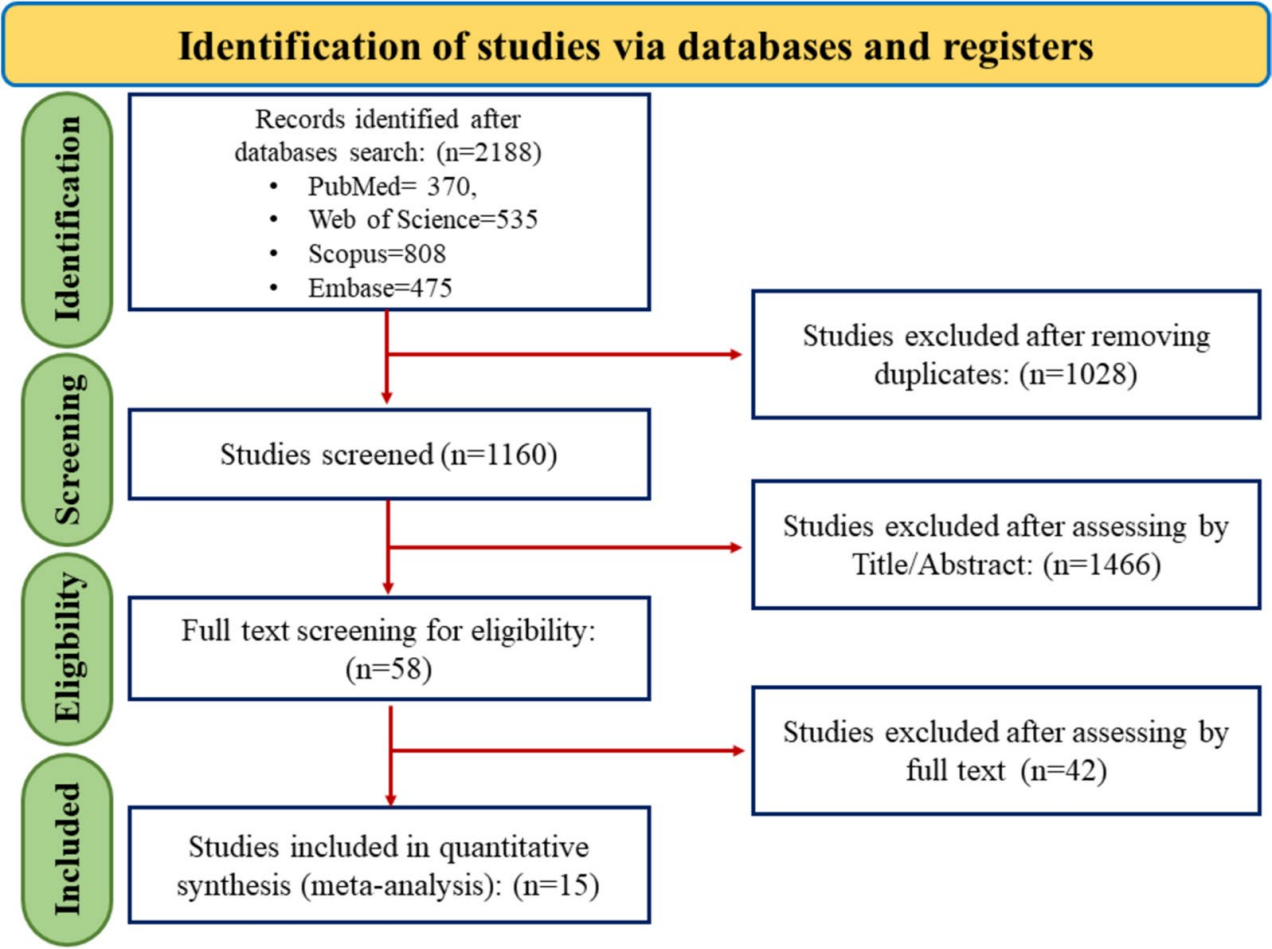
PRISMA flow diagram of the study selection process

The initial electronic search yielded 329 studies, of which 162 were excluded due to duplicate titles. Then, 145 of the 167 studies were excluded because of unrelated topics. The remaining 22 studies were retrieved for further full-text evaluations, and seven were excluded, mainly due to insufficient or missing data. Finally, 15 studies were selected for the systematic review, all used in the final meta-analysis. The details regarding the literature search and study selection process are presented in Fig. 2.

### Description of the included studies

Twelve RCTs selected included diabetic and pre-diabetic participants. All RCTs were published between 2015 and 2023; eleven RCTs were performed in Iran and one in Greece. The duration of the intervention with saffron ranged from 8 to 12 weeks. Ten studies used saffron supplementation, one used crocin supplementation, and one used both (crocin and saffron) for intervention. The dosage used for saffron intervention was between 15 and 1000 mg/day, and that for crocin was between 15 and 30 mg/day.

The characteristics of the included trials are listed in Table 2.

**Table 2:** Basic characteristics of included studies in meta-analysis

Author/References	Year	Study design	Population	Participants (Int./Cont.)	Gender (males/females)	Intervention Age (Int./Cont.)	Intervention	Dose (gr/day)	Duration
Azimi et al. [16]	2014	RCT Single-blind	T2DM	81 (42/39)	Males: 31 Females: 50	Int.: 57.02 (± 6.48) Cont.: 53.64 (± 8.11)	Saffron	1000	8 weeks
Milajerdi et al. [51]	2017	RCT Double-blind	T2DM	52 (26/26)	Males: 12 Females: 40	Int.: 54.57 (± 9.96) Cont.: 55.42 (± 7.58)	Saffron	30	8 weeks
Sepahi et al. [52]	2018	RCT Double-blind	T1DM T2DM	40 (20/20)	Males: 23 Females: 17	Int.: 56.09 (± 4.3) Cont.: 57.17 (± 2.9)	Crocin	15	12 weeks
Moravej aleali et al. [53]	2019	RCT Double-blind	T2DM	64 (32/32)	Males: 19 Females: 45	Int.: 53.5 (± 9.9) Cont.: 52.4 (± 13)	Crocus-sativus	30	12 weeks
Ebrahimi et al. [54]	2019	RCT Double-blind	T2DM	80 (40/40)	Males: 36 Females: 44	Int.: 55.2 (± 7.3) Cont.: 53 (± 10.6)	Saffron	100	12 weeks
Karimi-Nazari et al. [55]	2019	RCT Double-blind	Pre-DM	75 (36/39)	Males: 36 Females: 39	Int.: 57.95 (± 8.12) Cont.: 57.9 (± 8.7)	Crocus-sativus	15	8 weeks
Mobasseri et al. [56]	2020	RCT Double-blind	T2DM	57 (30/27)	None-Reported	Int.: 50.57 (± 9.88) Cont.: 51.63 (± 11.3)	Saffron	100	8 weeks
Behrouz et al. [57]	2020	RCT Double-blind	T2DM	50 (25/25)	Males: 7 Females: 43	Int.: 57.08 (± 7.41) Cont.: 59.86 (± 9.46)	Crocin	30	12 weeks
Tajaddini et al. [46]	2021	RCT Double-blind	T2DM	60 (30/30)	Males: 28 Females: 32	Int.: 50.5 (± 9.8) Cont.: 51.8 (± 10.9)	Saffron	100	8 weeks
Jaafarinia et al. [58]	2022	RCT Triple‑blind	T2DM	40 (21/19)	Males: 23 Females: 17	Int.: 63.86 (± 10.62) Cont.: 62.68 (± 9.84)	Crocin	15	12 weeks
Dastkhosh et al. [59]	2022	RCT Double-blind	T2DM	45 (23/22)	Males: 7 Females: 38	Int.: 57.08 (± 7.41) Cont.: 59.86 (± 9.46)	Crocin	30	12 weeks
Rajabi et al. [60]	2022	RCT	T2DM	16 (8/8)	Males: 0 Females: 16	Int.: 54.12 (± 7.37) Cont.: 56.87 (± 5.11)	Saffron	400	8 weeks
Sepahi et al. [18]	2022	RCT Triple‑blind	T2DM	100 (50/50)	Males: 46 Females: 54	Int.: 57.16 (± 1.5) Cont.: 56.92 (± 1.9)	Saffron	30	12 weeks
Kotanidou et al. [19]	2023	RCT Double-blind	Pre-DM	49 (25/24)	Males: 23 Females: 26	Int.: 12.32 (± 1.43) Cont.: 12.27 (± 1.87)	Crocus-sativus	60	12 weeks
Tajaddini et al. [17]	2023	RCT Double-blind	T2DM	60 (30/30)	Males: 28 Females: 32	Int.: 50.57 (± 9.88) Cont.: 51.83 (± 10.91)	Crocus-sativus	100	8 weeks

RCT: randomized clinical trial; SB: Single-blind; DB: double-blind; T1DM: Type 1 Diabetes mellitus; T2DM: Type 2 Diabetes mellitus; Int.: Intervention; Cont.: Control; NR: None-Reported

^a^Reported by (mean-SD)

### Study characteristics

Of the 15 included RCTs, most (n = 14) were from Iran, and only one was from Greece. The RCTs included were published between 2014 and 2023. Ten of these studies were performed on patients with T2DM, one with type 1 and type 2 DM, and 3 in patients with T2DM, overweight and obese. In these 15 studies, we extracted data from 869 participating patients. The demographics and features of studies and patients are gathered in Table 2. The sample size ranged from 16 to 100 participants.

### Effect of saffron on FBS

**Fig. 3.**
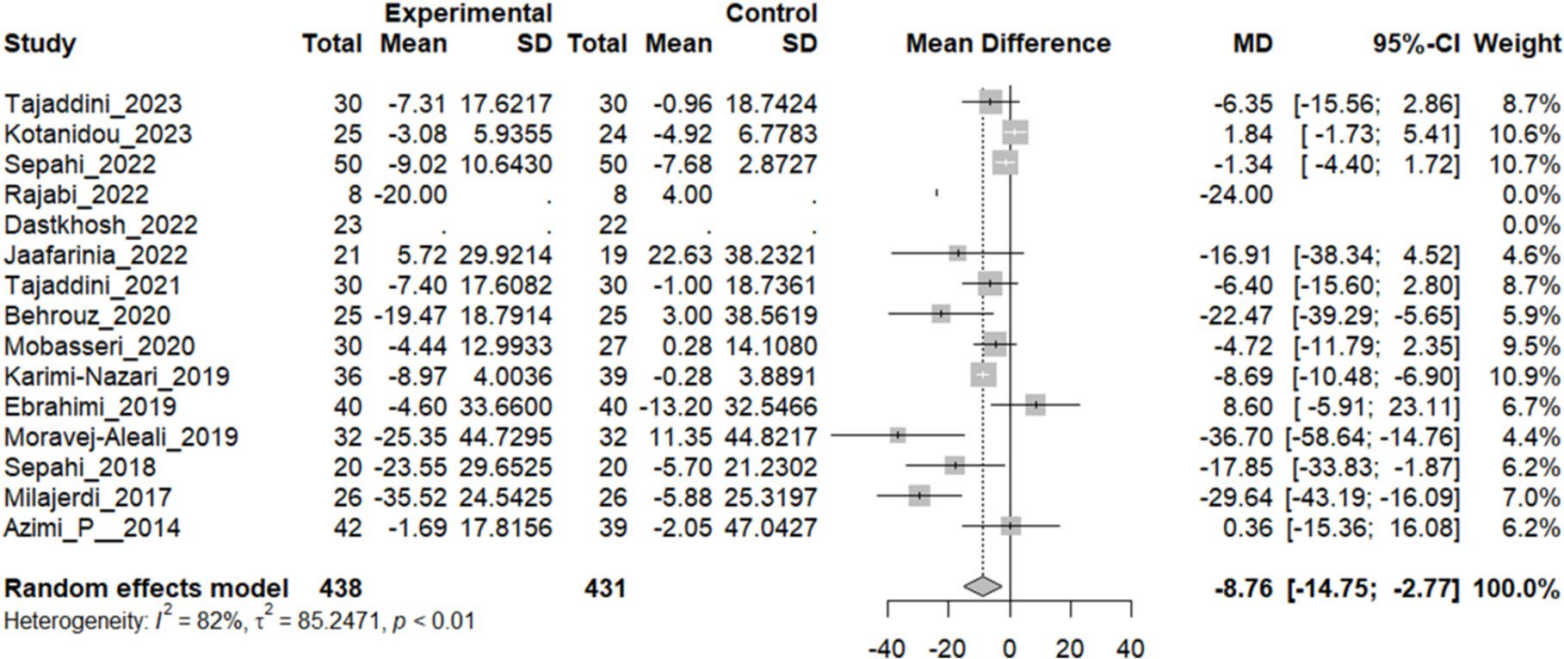
13 studies reported a pooled meta-analysis of the mean difference in FBS between the intervention and control groups

Thirteen studies reported a change in FBS mean in patients who received the intervention and control. The overall pooled analysis of 13 studies showed an MD of − 8.75 mg/dL, 95% CI [− 14.75; − 2.76], P-value < 0.01. Therefore, the difference between the intervention and the control group was statistically significant in favor of the intervention group. Ten studies showed a decline in the mean of FBS; the highest decline in the mean of the patient’s FBS was reported by Moravej-Alaei 2019; MD: − 36.70 mg/dL, 95%CI [− 58.64, − 14.76]. However, two studies showed a converse effect and increased mean FBS in the control group. The highest increase was seen in a survey conducted by Ebrahimi. et al. in 2019 MD: 8.60 mg/dL, [95% CI − 5.91 to 23.11]. The heterogeneity among these studies was statistically significant (I^2^ = 82, Q (12):66.74, and P-value < 0.01) (Fig. 3).

### Effect of Saffron on HbA1C

**Fig. 4:**
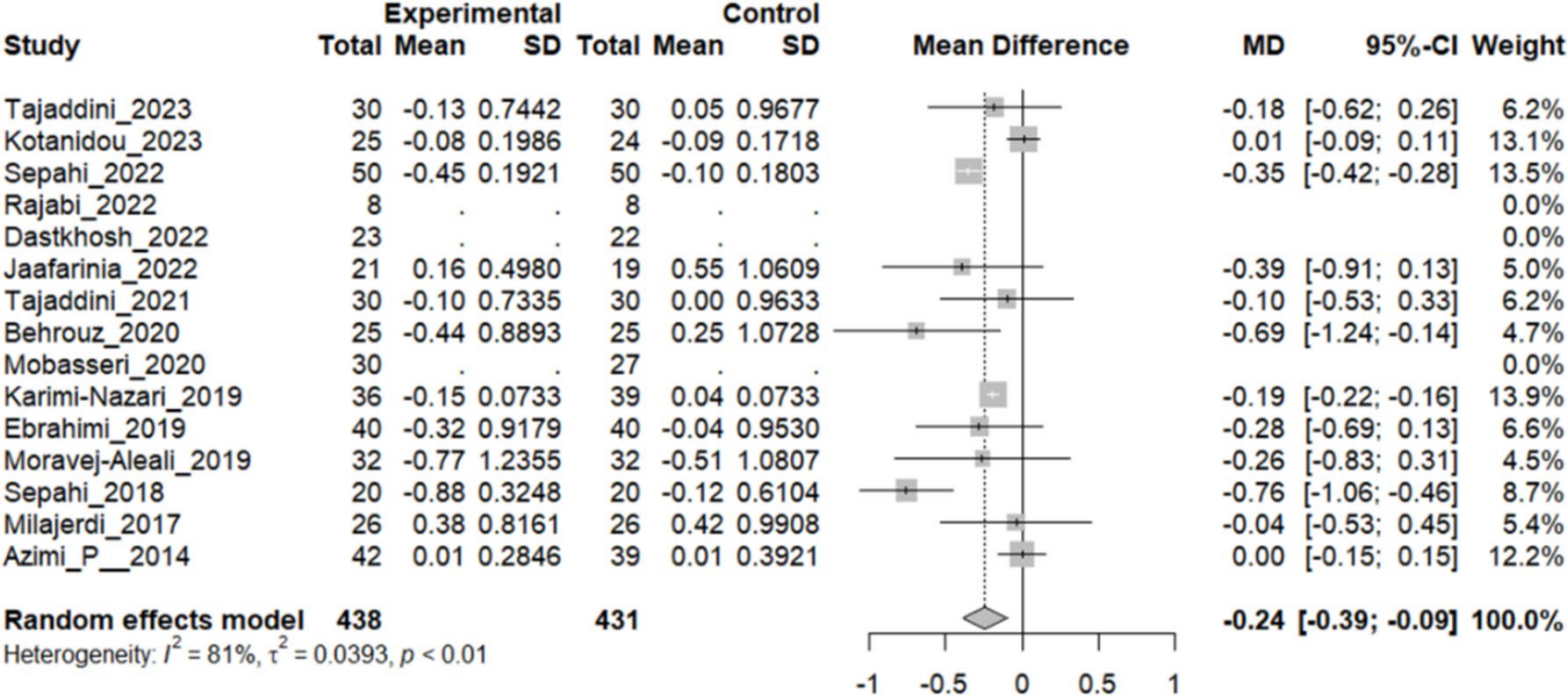
12 studies reported a pooled meta-analysis of the mean difference in HbA1C between the intervention and control groups

Twelve studies reported a change in the mean of HbA1C in patients who received the intervention and control. The overall pooled analysis of 12 studies showed an MD of − 0.34%, 95% CI [− 0.39; − 0.9], and P-value < 0.01. Therefore, the difference between the intervention and the control group was statistically significant in favor of the intervention group. Ten studies showed a decline in the mean HB A1C; the highest drop in the mean of the patient’s HbA1C was reported by Sepahi et al. [52]; MD: − 0.76%, 95%CI [− 1.06, − 0.46]. However, two studies showed no difference in the mean of HB A1C between both groups. The heterogeneity among these studies was statistically significant (I^2^ = 81%, Q (11):56.59, and P-value < 0.01) (Fig. 4).

### Effect of saffron on insulin and HOMA

**Fig. 5:**
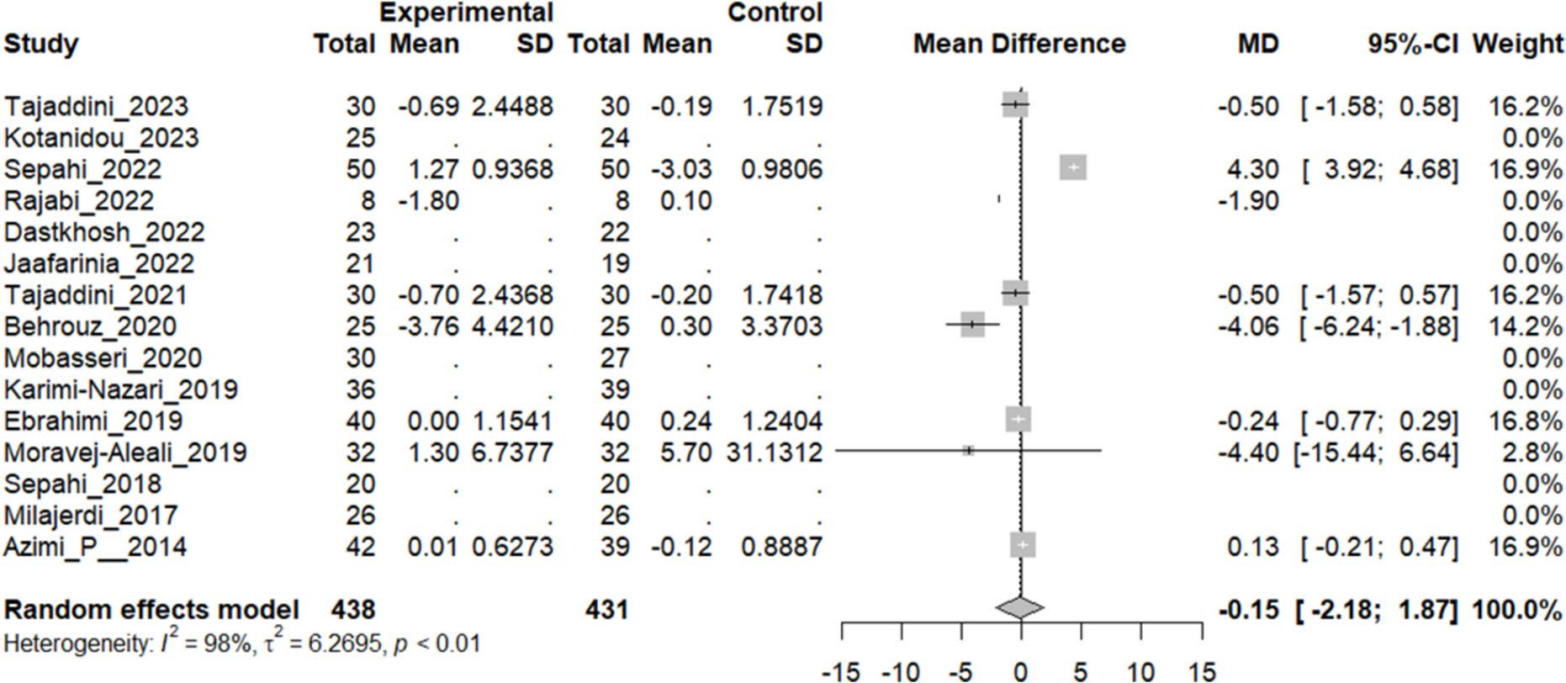
Seven studies reported a pooled meta-analysis of the mean difference in insulin dose between the intervention and control groups

**Fig. 6:**
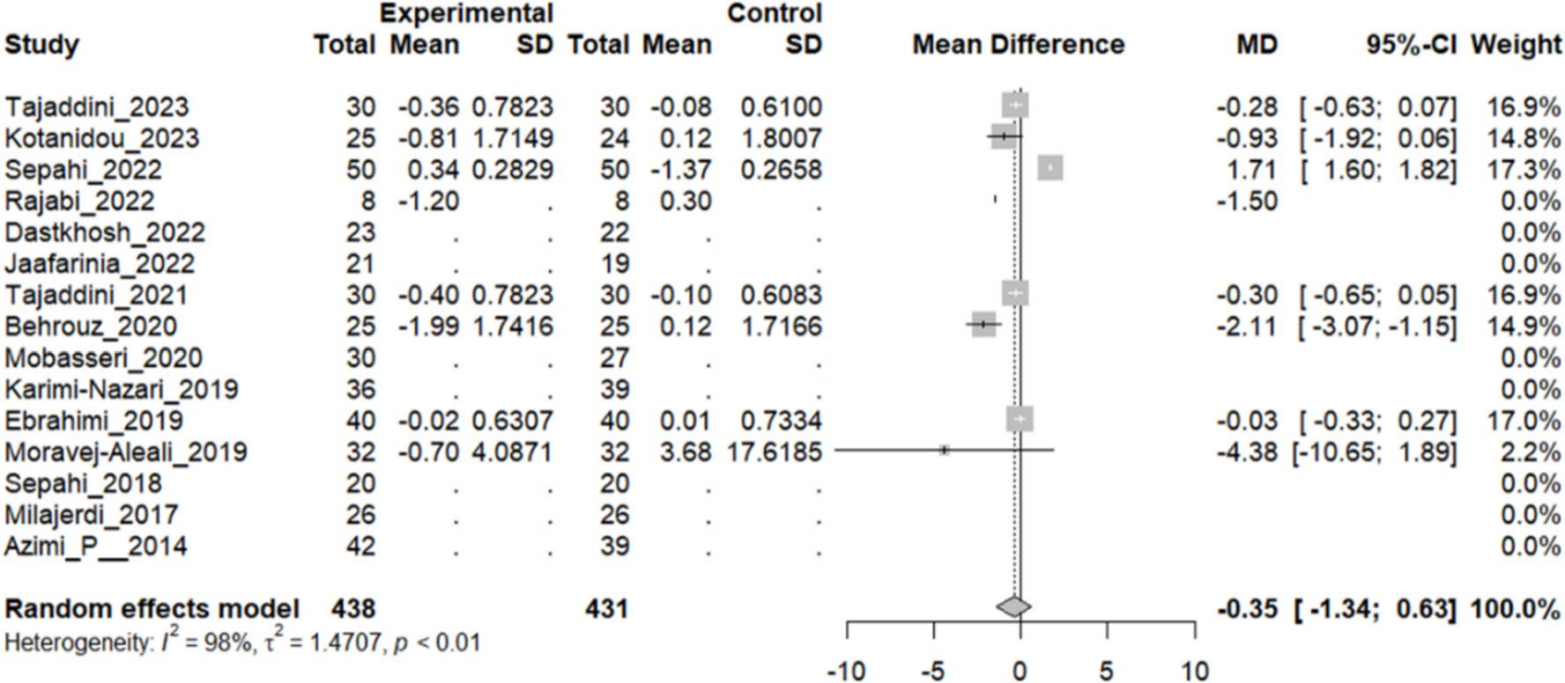
Studies reported a pooled meta-analysis of the mean difference in HOMA between the intervention and control groups

Seven studies evaluated the insulin dose difference between the intervention and control group and HOMA, an indicator of insulin resistance. Although the pooled analysis has shown a decline in both indicators, the difference between the mean of insulin secretion (MD: − 0.15, 95% CI [− 2.1763; 1.8689], P-value: 0.88) (Fig. 5) and HOMA (MD: − 0.35, 95% CI [− 1.34; 0.63], P-value: 0.48) (Fig. 6) was not statistically significant.

### Effect of saffron on triglyceride (TG)

**Fig. 7:**
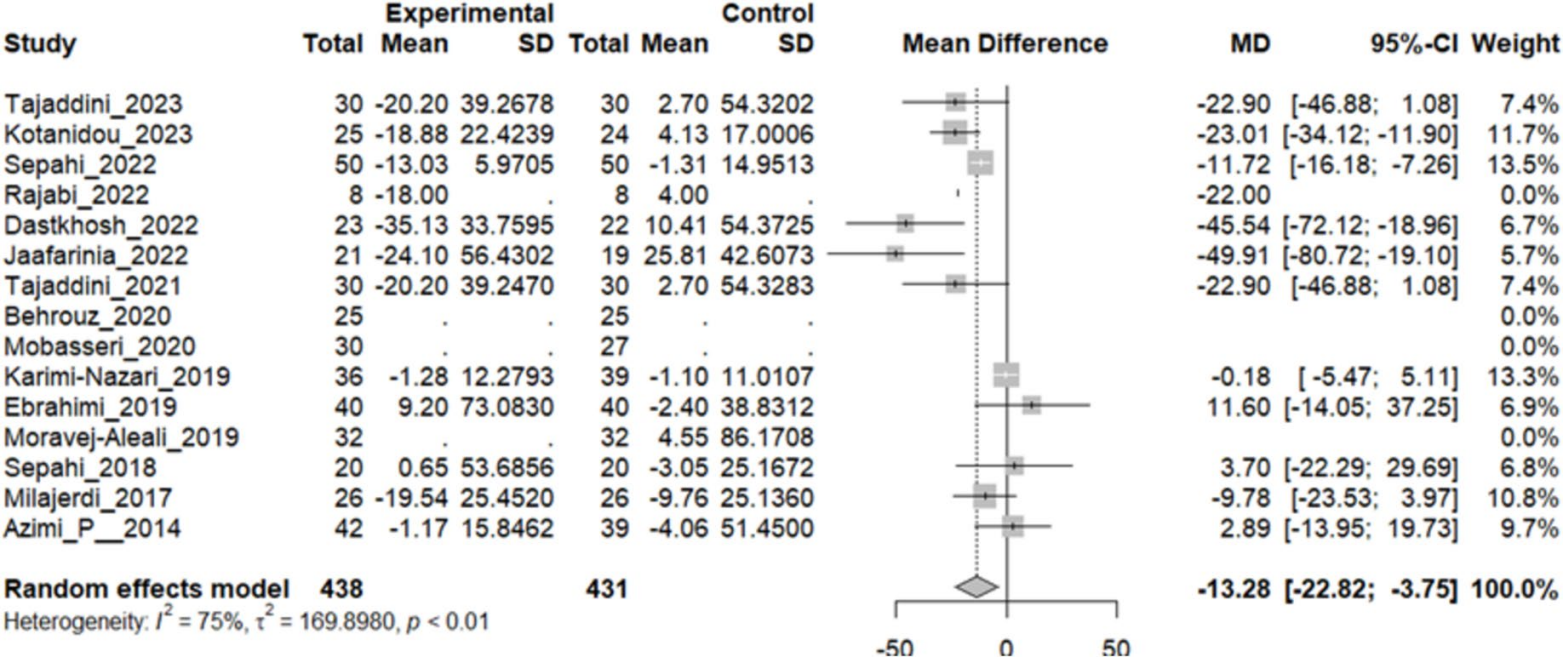
Eleven studies reported a pooled meta-analysis of the mean difference in TG between the intervention and control groups

Eleven studies reported a change in the mean of TG in patients who received the intervention and control. The overall pooled analysis of 12 studies showed an MD of − 13.28 mg/dL, 95% CI [− 22.82; − 3.75], P-value < 0.01. Therefore, the difference between the intervention and the control group was statistically significant in favor of the intervention group (Fig. 7).

### Effect of saffron on total cholesterol (TC)

**Fig. 8:**
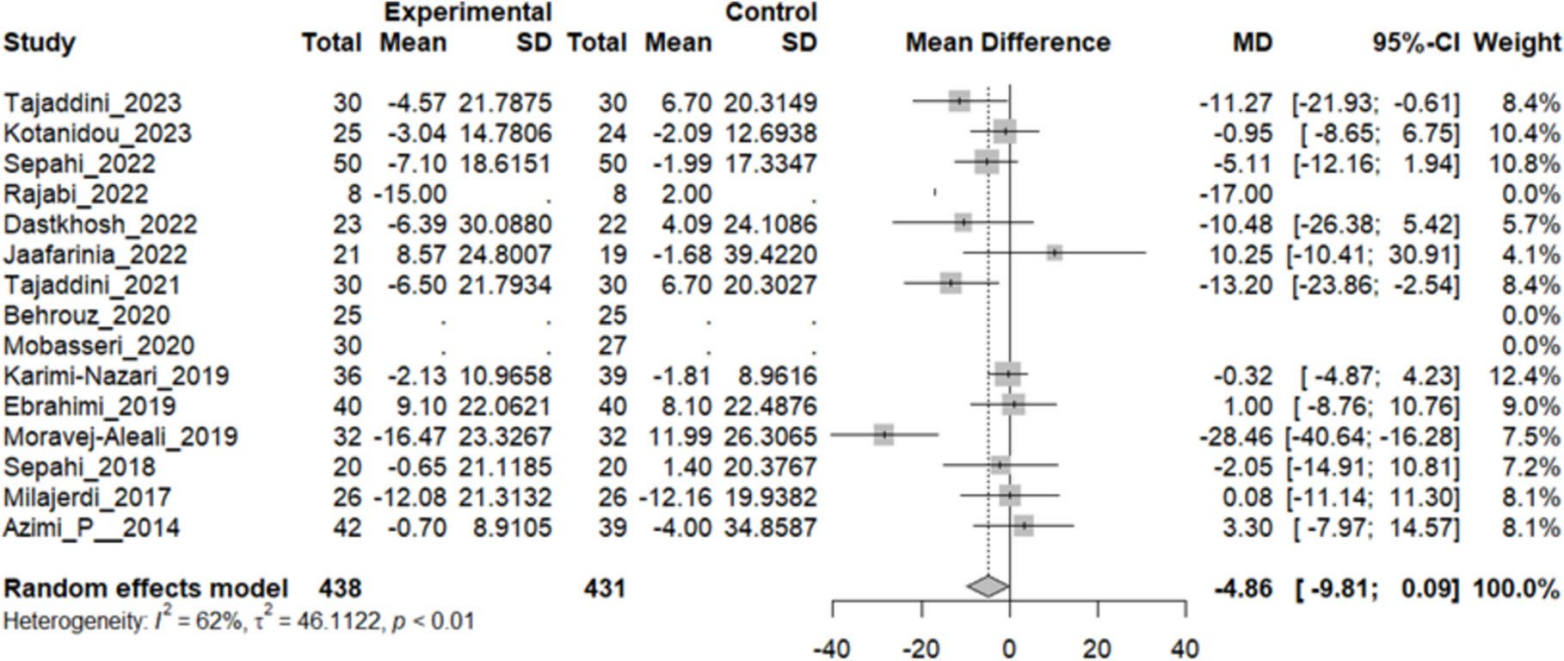
Twelve studies reported a pooled meta-analysis of the mean difference in TC between the intervention and control groups

Twelve studies reported a change in the mean TC in patients who underwent the intervention compared to the control group. The overall pooled analysis of 12 studies showed an MD of − 4.86 mg/dL, 95% CI [− 9.81 to 0.09], and a P-value of 0.54. Therefore, the difference between the intervention and the control group was not statistically significant. The heterogeneity among these studies was statistically significant (I^2^ = 62%, Q (11): 28.93, and P-value: 0.01) (Fig. 8).

### Effect of saffron on HDL

**Fig. 9:**
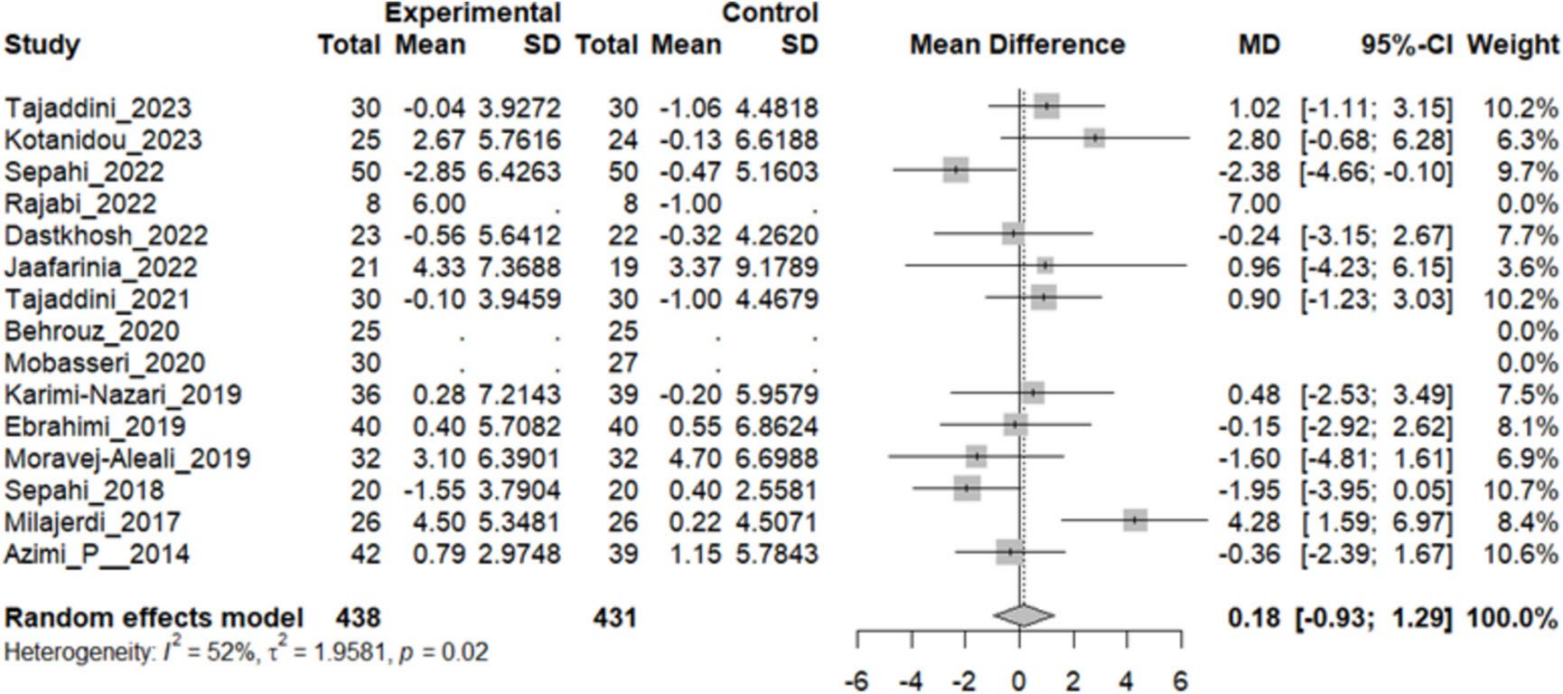
Twelve studies reported a pooled meta-analysis of the mean difference in HDL between the intervention and control groups

Twelve studies reported a change in the mean HDL in patients who underwent the intervention compared to the control group. The overall pooled analysis of 12 studies showed an MD of 0.18 mg/dL, 95% CI [− 0.93; 1.29], and a P-value of 0.74. Therefore, the difference between the intervention and the control group was not statistically significant. The heterogeneity among these studies was statistically significant (I^2^ = 52%, Q [11]: 22.91, and P-value: 0.02) (Fig. 9).

### Effect of saffron on LDL

**Fig. 10:**
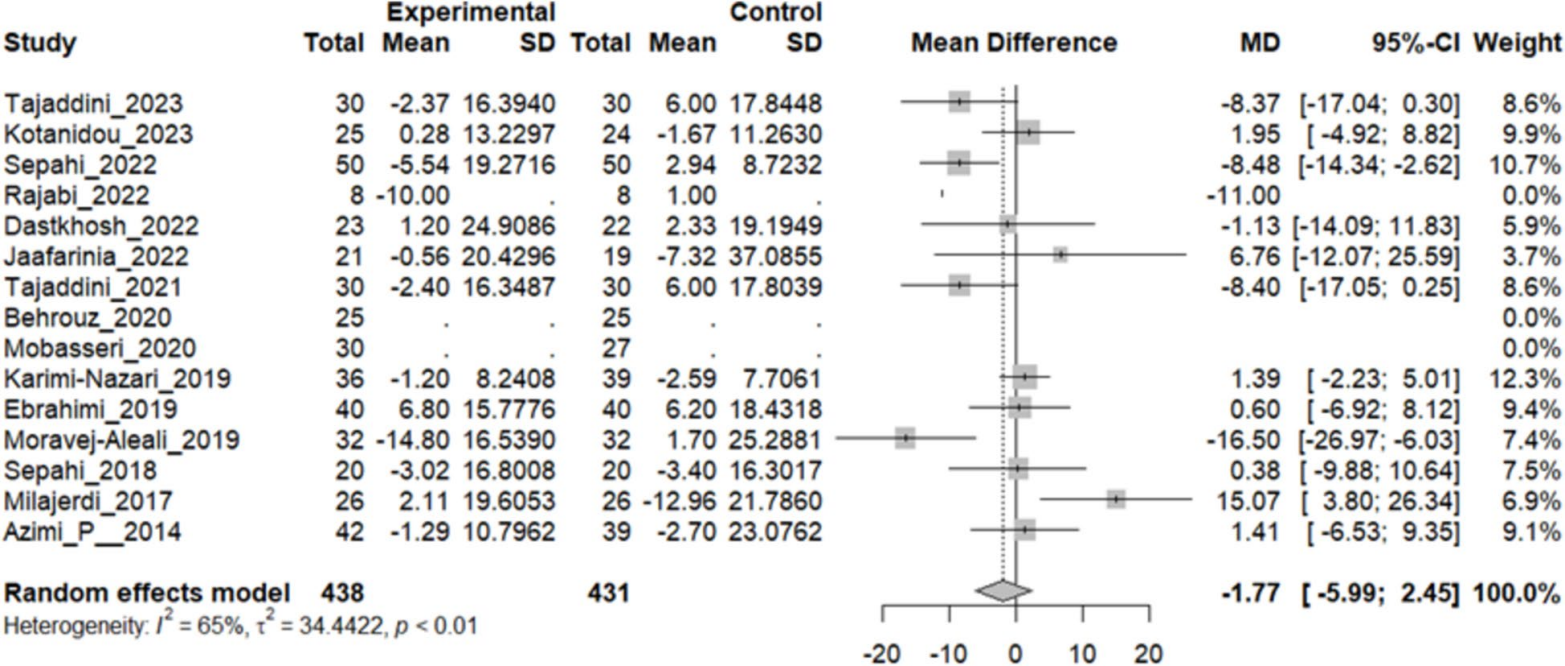
Twelve studies reported a pooled meta-analysis of the mean difference in LDL between the intervention and control groups

Twelve studies reported a change in the mean LDL in patients who underwent the intervention compared to the control group. The overall pooled analysis of 12 studies showed an MD of − 1.77 mg/dL, 95% CI [− 5.99–2.45], and a P-value of 0.41. Therefore, the difference between the intervention and the control group was not statistically significant. The heterogeneity among these studies was statistically significant (I^2^ = 65%, Q (11): 31.58, and P-value: < 0.01) (Fig. 10).

#### Effect of saffron on blood pressure

**Fig. 11:**
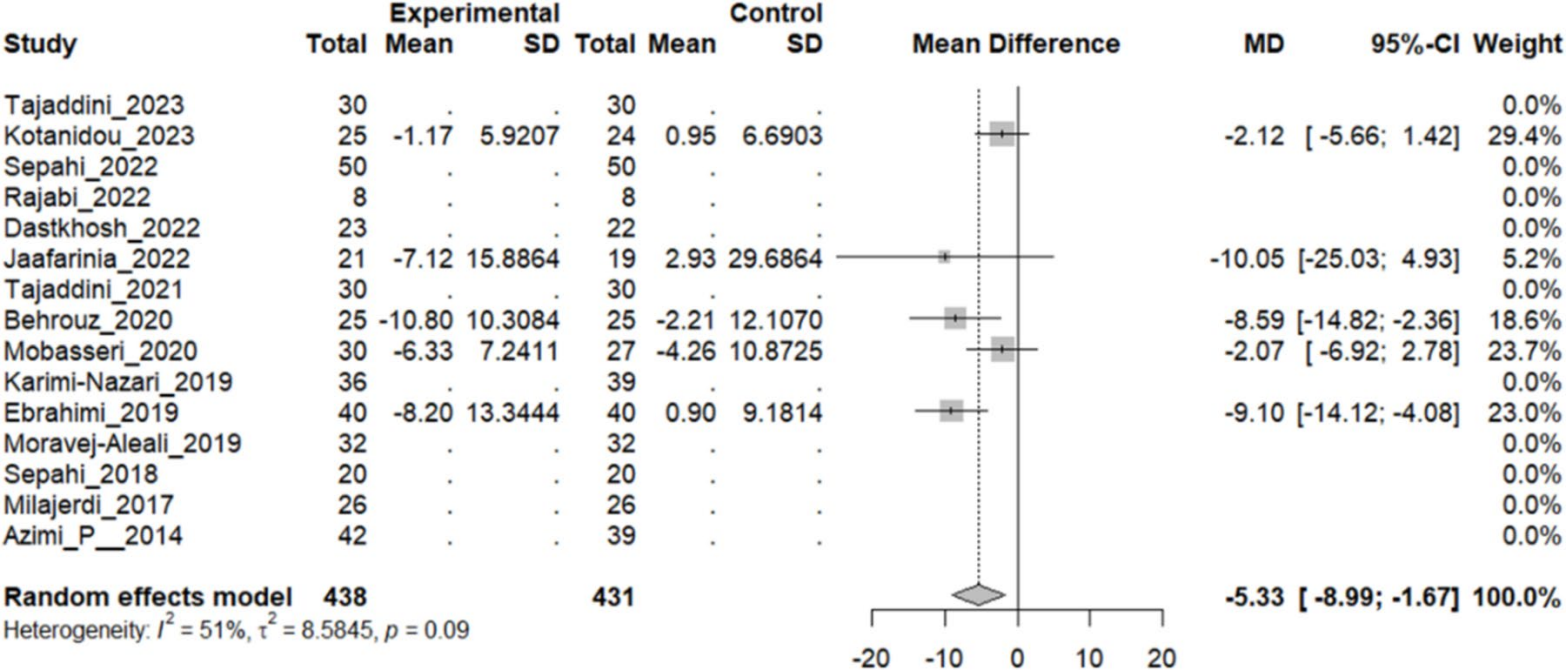
Five studies reported a pooled meta-analysis of the mean difference in SBP between the intervention and control groups

Five studies reported a change in the mean of systolic blood pressure (SBP) in patients who underwent the intervention compared to the control group. They all concluded that the mean would be lower in the intervention group. The overall pooled analysis of 5 studies showed an MD of − 5.33 mmHg, 95% CI [− 8.99 to 1.67], and a P-value of 0.04. Therefore, the difference between the intervention and the control group was statistically significant. The heterogeneity among these studies was not statistically significant (I^2^ = 51%, Q (4): 8.10, and P-value: 0.08) (Fig. 11).

**Fig. 12:**
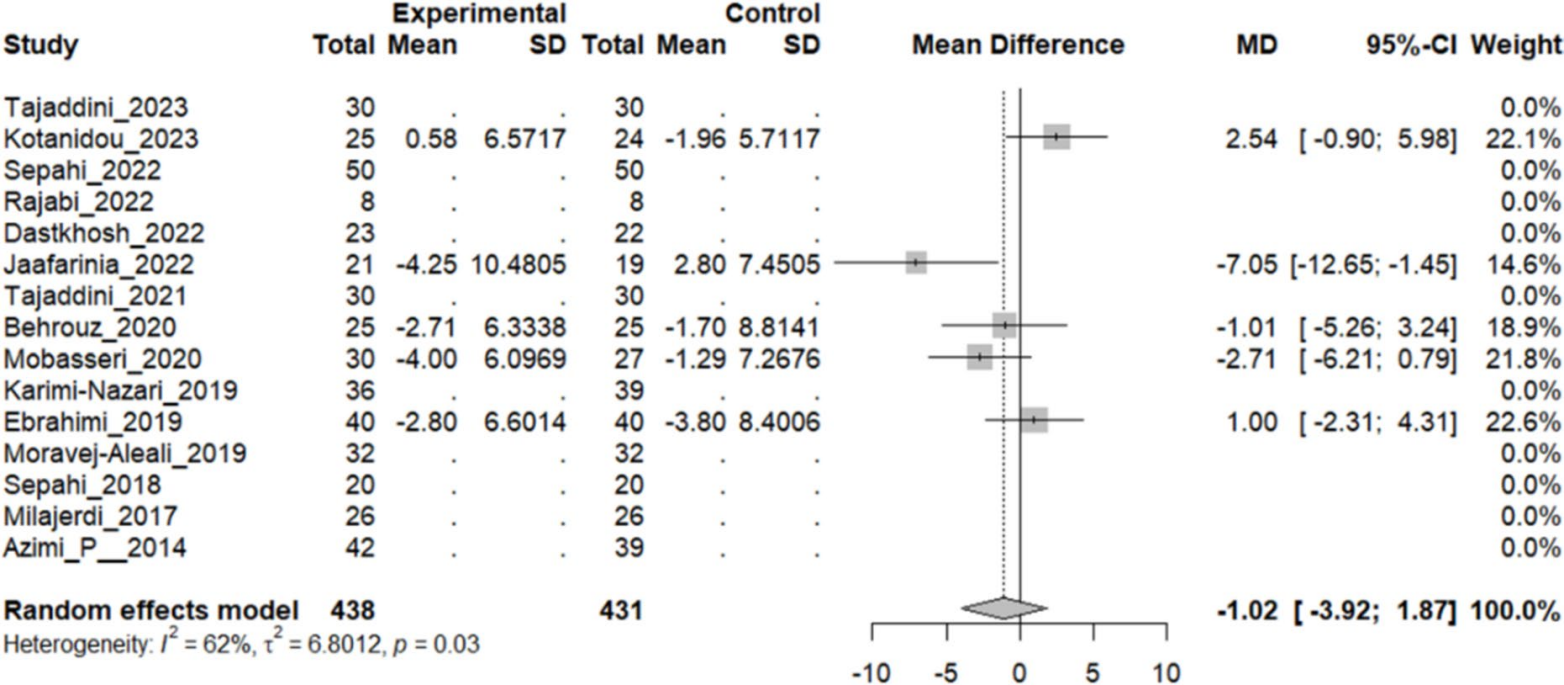
Five studies reported a pooled meta-analysis of the mean difference in DBP between the intervention and control groups

On the other hand, four studies evaluated the diastolic blood pressure (DBP) average, which showed a reduction of MD in all of them. The most prominent one was concluded by *Jafarnia* et al. in 2022 with a decrease of DBP: − 7.05, 95% CI [− 12.6461; − 1.4539]. The pooled analysis, however, demonstrated a reduction of − 1.02 mmHg, 95% CI [− 3.91; 1.86], P-value: 0.4878. Therefore, the difference between the two groups was statistically insignificant. The heterogeneity among these studies was statistically significant (I2 = 62%, Q [4]: 10.62, and P-value: 0.03) (Fig. 12).

#### Effect of Saffron on Aspartate aminotransferase (AST)

**Fig. 13:**
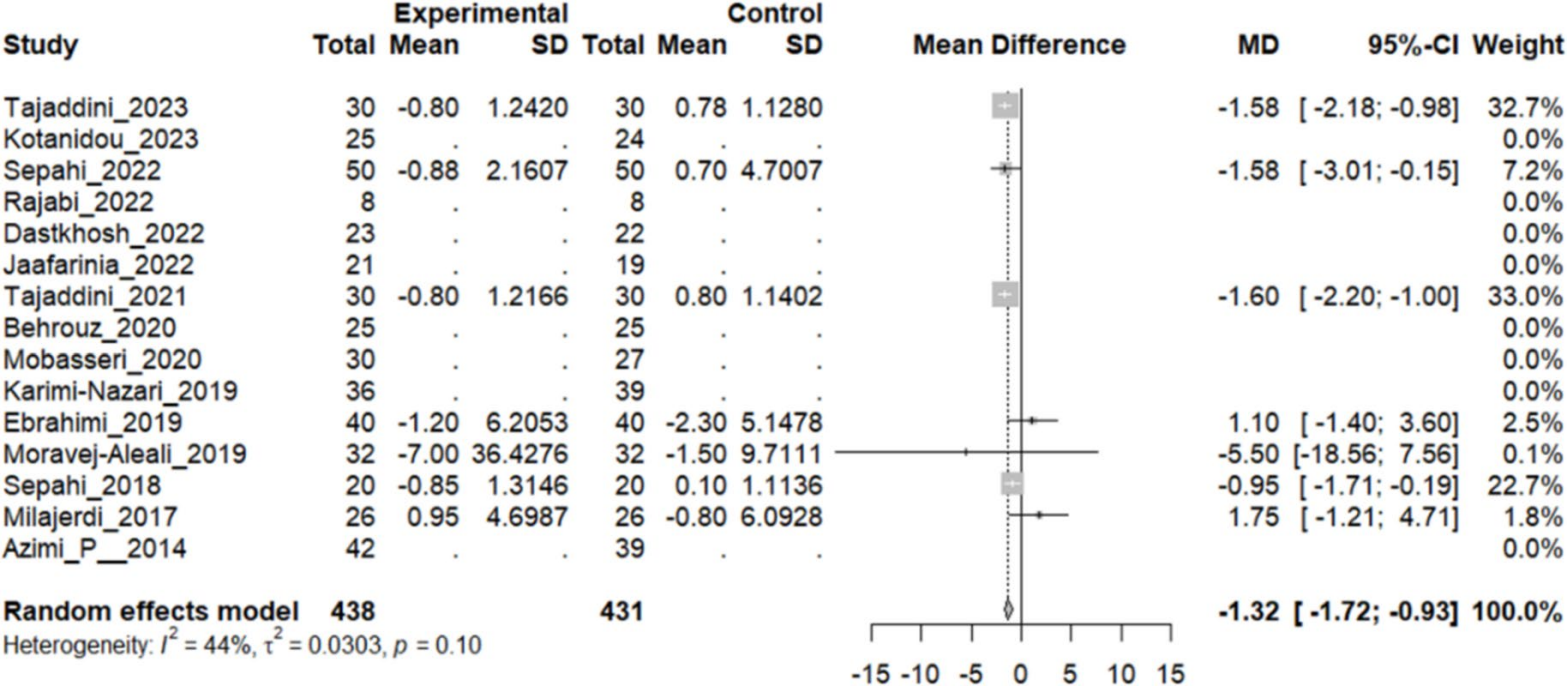
Seven studies reported a pooled meta-analysis of the mean difference in the blood level of AST between the intervention and control groups

Seven studies reported a change in the mean AST level in patients who underwent the intervention compared to the control group. Except for two studies, others concluded that the mean AST levels in the intervention group were lower than in the control group. The overall pooled analysis of seven studies showed an MD of − 1.32 mmHg, 95% CI [− 1.72, − 0.93], and a P-value of < 0.01. Therefore, the difference between the intervention and the control group was statistically significant. The heterogeneity among these studies was not statistically significant (I^2^ = 44%, Q (6): 10.72, and P-value: 0.1) (Fig. 13).

#### Effect of saffron on alanine aminotransferase (ALT)

**Fig. 14:**
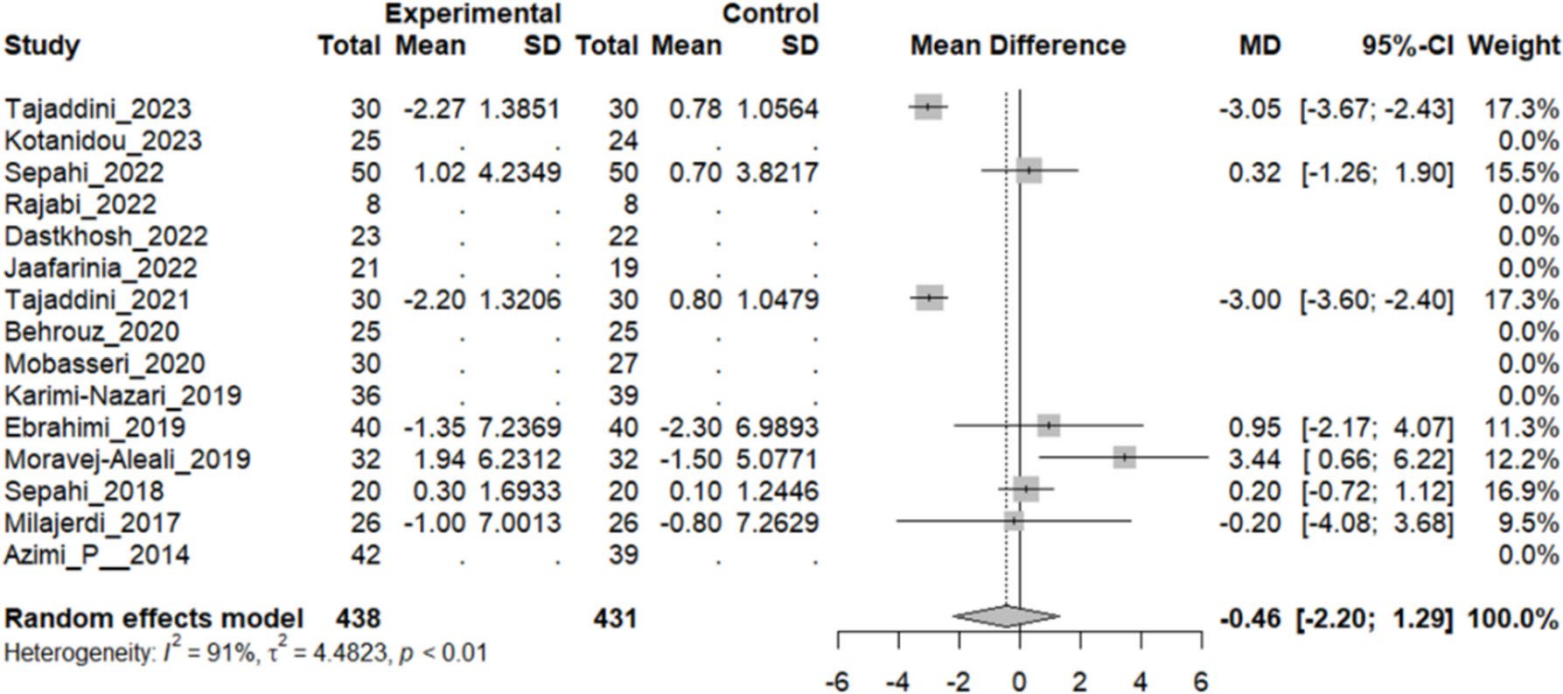
Seven studies reported a pooled meta-analysis of the mean difference in ALT blood levels between the intervention and control groups

Seven studies reported a change in the mean ALT level in patients who underwent the intervention compared to the control group. Except for two studies (Tajdini 2021 and 2023), which showed a significant decline in ALT levels in the intervention group, and Moravej_Alaei’s study that showed increased ALT levels in intervention, others demonstrated no significant difference in the blood level of this liver enzyme. The overall pooled analysis of seven studies showed an MD of − 0.46 mmHg, 95% CI [− 2.20, 1.29], and a P-value of 0.60. Therefore, the difference between the intervention and the control group was not statistically significant. The heterogeneity among these studies was statistically significant (I^2^ = 91%, Q (6): 70.23, and P-value < 0.1) (Fig. 14).

#### Effect of saffron on creatinine (Cr)

**Fig. 15:**
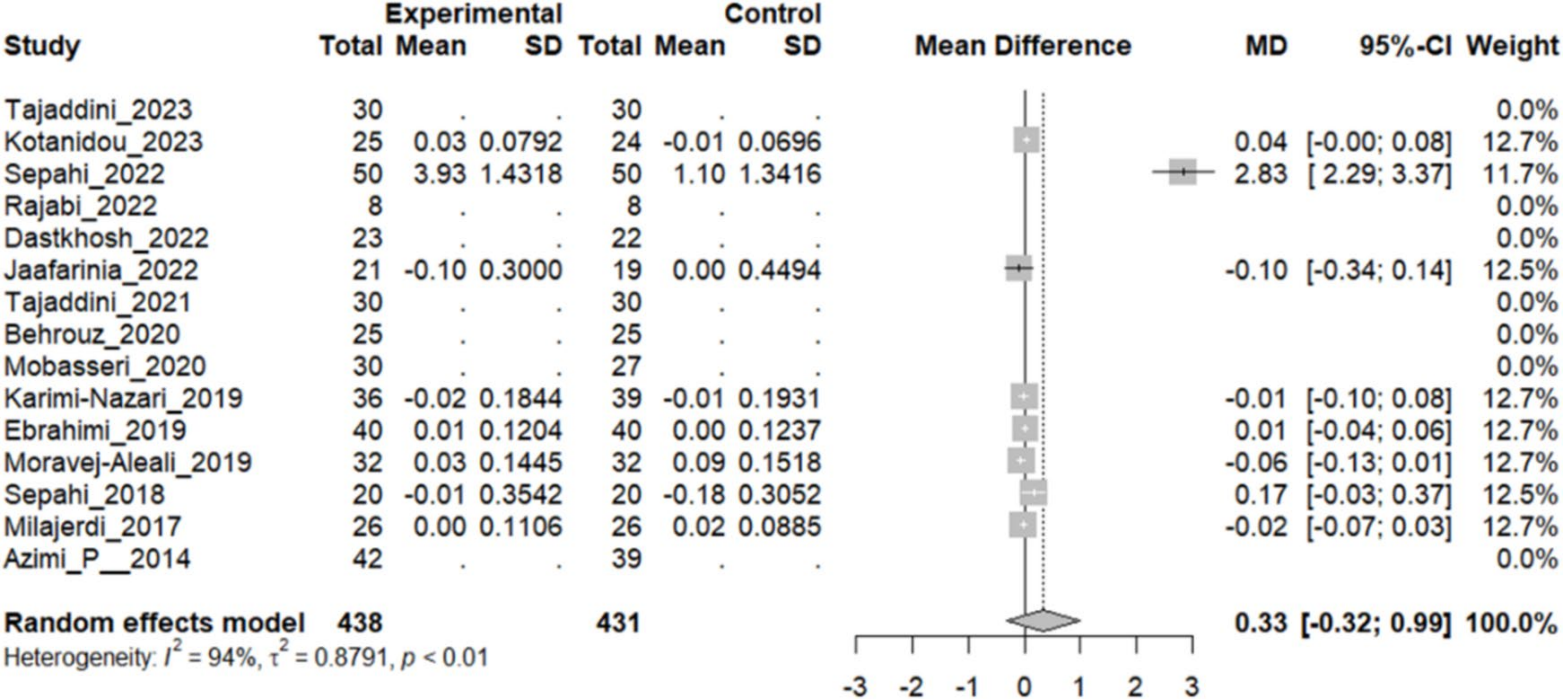
Eight studies reported a pooled meta-analysis of the mean difference in Cr blood level between the intervention and control groups

Eight studies reported a change in the mean creatinine level in patients who underwent the intervention compared to the control group. While four studies showed a negligible decline in the creatinine level in the intervention group, three others showed a minimal increase in patients who had taken saffron. However, a survey was conducted by Sepahi et al. [18] showed a significant increase in the mean creatinine level (MD: 2.83 mg/dL, 95% CI [2.29, 3.37]). The overall pooled analysis of 7 studies showed an MD of 0.33 mg/dL, 95% CI [− 0.32, 0.99], and a P-value of 0.31. Therefore, the difference between the intervention and the control group was statistically insignificant. The heterogeneity among these studies was not statistically significant (I^2^ = 94%, Q (7): 113.35, and P-value < 0.1) (Fig. 15).

#### Effect of saffron on blood urea nitrogen (BUN)

**Fig. 16:**
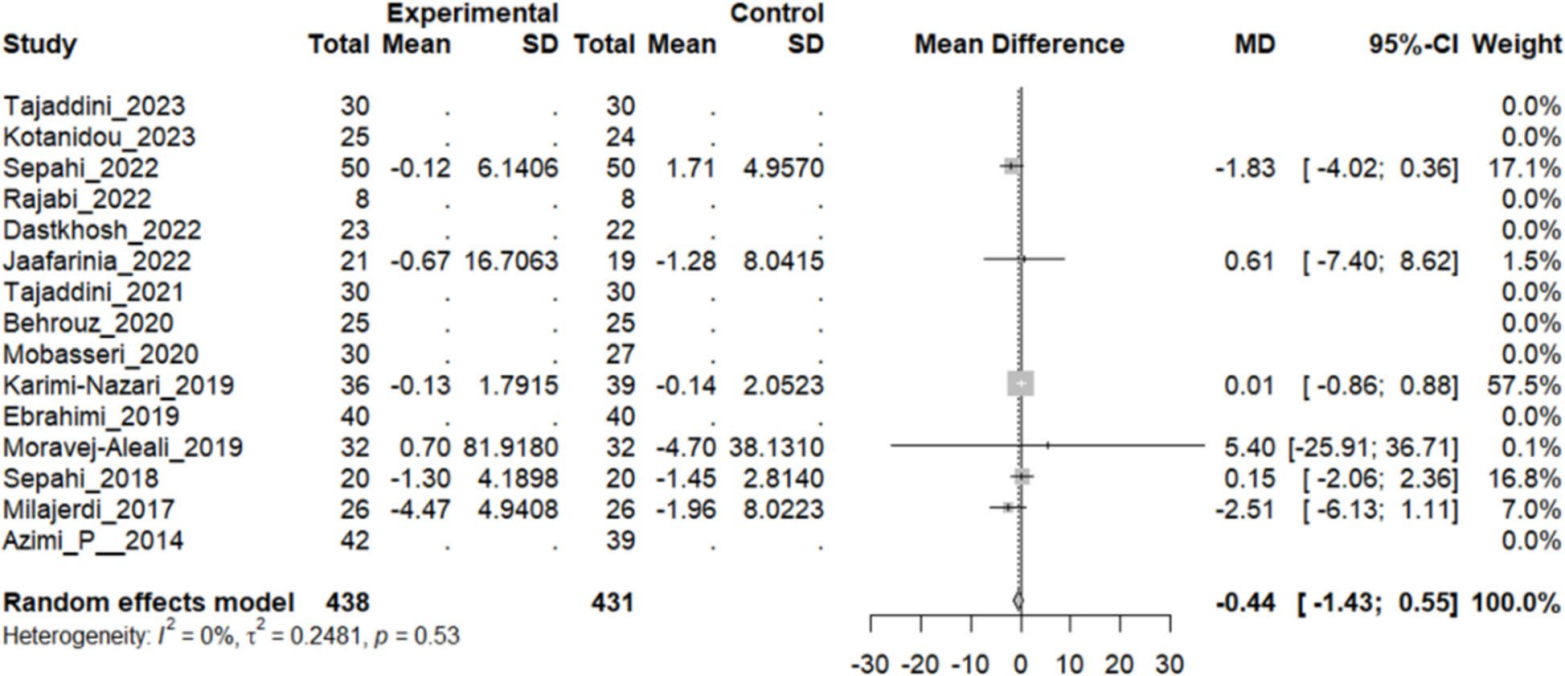
Six studies reported a pooled meta-analysis of the mean difference in blood BUN levels between the intervention and control groups

Six studies reported a change in the mean BUN level in patients who underwent the intervention compared to the control group. While two studies showed a significant decline in the BUN level in the intervention group, another study performed by Moravej-Alaei et al. showed a considerable increase in BUN level (MD: 5.40 mg/dL, 95% CI [− 25.91, 36.71]). The overall pooled analysis of seven studies showed an MD of − 0.44 mg/dL, 95% CI [− 1.43, 0.55], and a P-value of 0.38. Therefore, the difference between the intervention and the control group was not statistically significant. The heterogeneity among these studies was not statistically significant (I^2^ = 0%, Q (5): 4.13, and P-value 0.53) (Fig. 16).

#### Tumor necrosis factor-alpha (TNF-α)

**Fig. 17:**
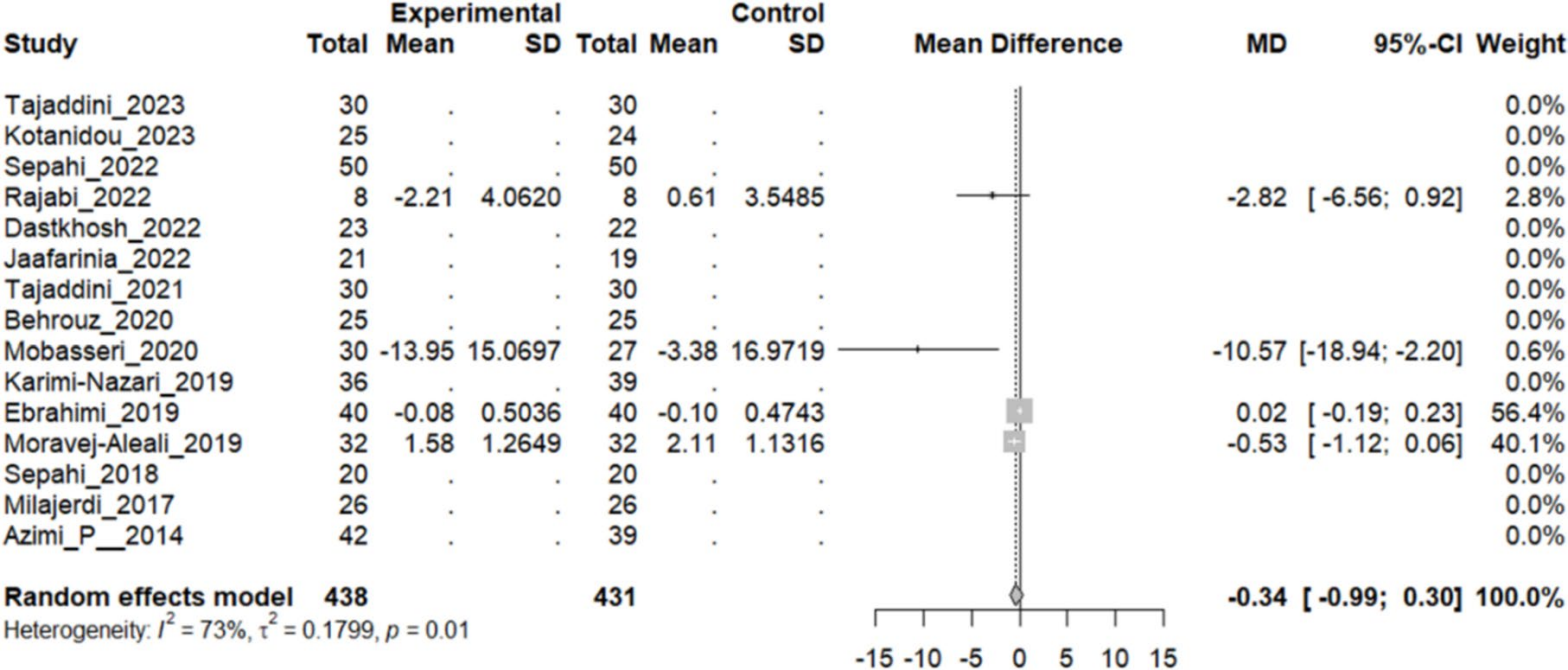
Four studies reported a pooled meta-analysis of the mean difference in TNF-α between the intervention and control groups

Four studies reported a change in the mean TNF-α level in patients who underwent the intervention compared to the control group. However, three studies showed a decrease in the mean of this inflammatory marker in the intervention group, Ebrahimi et al. concluded that there was no considerable change in the MD of TNF-α level. The overall pooled analysis of 4 studies showed an MD of − 0.34 pg/mL, 95% CI [− 0.99–0.30], and a P-value of 0.29. Therefore, the difference between the intervention and the control group was not statistically significant. The heterogeneity among these studies was statistically significant (I2 = 73%, Q(3): 11.14, and P-value: 0.01) (see Fig. 17).

#### Publication *bias*

Egger's test and funnel plots were used to determine the publication bias in this systematic review and meta-analysis. There was a considerable and significant publication for the mean difference of FBS (P = 0.02), HbA1C (P < 0.01), HOMA (P = 0.02), SBP (P < 0.01), DBP (P = 0.03), TNF-α (P ≤ 0.01), ALT (P < 0.01), and creatinine (P < 0.01). However, a statistically significant publication bias was not detected in the mean difference estimated for insulin dose (P = 0.14), TG (P = 0.23), HDL (P = 0.37), TC (P = 0.84), LDL (P = 0.59), AST (P = 0.09), and BUN (P = 0.67). The funnel plots are present in supplementary materials.

## Discussion

Our systematic review and meta-analysis study evaluated the effects of saffron and its extract (crocin) on various cardiometabolic indices in diabetic and prediabetic overweight. Our findings revealed that saffron significantly reduced FBS, HbA1C levels, TG, and SBP, AST. However, no significant effects were observed on HDL, total cholesterol, LDL, insulin secretion, HOMA, DBP, TNF, ALT, creatinine, or BUN.

In alignment with our study, another recent meta-analysis evaluated saffron's impact on glycemic control in diabetes patients and showed saffron (5 mg/day to 1 g/day) significantly reduced fasting plasma glucose (FPG) compared to placebo. However, it had no significant effect on insulin levels, QUICKI, or HOMA-IR [24]. FBS and HbA1C are critical indicators used in the management and diagnosis of diabetes [25, 26]. Although the hyperinsulinemic-euglycemic clamp is the gold standard for assessing insulin resistance due to its complexity, HOMA-IR is commonly used, mainly reflecting hepatic insulin resistance [27]. Our study showed that saffron significantly reduces FBS and HbA1C without affecting HOMA and insulin secretion, which was aligned with another study that showed saffron extract reduced HbA1c and FBS [28].

A linear relationship between the duration of saffron intake and reductions in FBG, as well as a non-linear dose–response relationship with HOMA-IR, FBG, and HbA1c, has been demonstrated in the study by Zamani et al. [29]. The primary ingredient in saffron is crocin, which is present as crocetin glycosides and serves as the main pigment in saffron. Crocin is notably responsible for many of saffron's recognized properties, such as its antidiabetic effects [30]. A study on alloxan-diabetic rats showed that saffron, particularly its constituents crocin, crocetin, and safranal, may reduce blood sugar levels by enhancing insulin levels and decreasing FBS and HbA1c. This anti-hyperglycemic effect could be potentially due to the antioxidant properties of crocetin, which is derived from the hydrolysis of orally administered crocin in the intestines [31]. Additionally, Crocetin could improve insulin sensitivity by regulating adiponectin, TNF-α, and leptin expression [32].

Previous studies have shown that certain supplements can improve glycemic control. Purslane significantly reduces FBSlevels but does not affect serum insulin, HOMA-IR, or QUICKI levels [33]. Probiotic supplementation significantly lowers fasting plasma glucose, HbA1c, HOMA-IR, and insulin levels, suggesting benefits for short-term use [34]. Moreover, a recent study found Cinnamon reduces FBS, HOMA-IR, and HbA1c in type 2 diabetes patients, though it does not significantly alter serum insulin levels [35].

Although our study revealed that Saffron significantly lowered TG, it did not significantly affect HDL, total cholesterol, or LDL levels. These results align with a previous study, indicating that saffron supplementation did not reduce lipid profiles [36]. In contrast with our study, Kazemi et al. demonstrated that saffron supplementation significantly reduced TG, total cholesterol, and LDL [31]. Crocin, a bioactive compound in saffron, reduces fat and cholesterol absorption by inhibiting pancreatic lipase [37]. In addition, Saffron, containing phytosterols and carotenoids, has been shown to lower LDL cholesterol, plasma TG, and overall cholesterol levels [38]. Also, a recent study explained that picrocrocin-enriched fraction from saffron stigmas (*Crocus sativus* L.) through a non-statin-like mechanism, effectively reducing lipid levels [39].

Saffron supplementation reduced SBP significantly with moderate and non-significant heterogeneity but did not show a statistically significant effect on DBP, which exhibits considerable heterogeneity. However, another study indicated that saffron supplementation significantly reduced both SBP and DBP, with a non-linear reduction in DBP concerning the duration of supplementation [40]. The chronic effects of saffron (*Crocus sativus L.*) stigma aqueous extract on blood pressure in normotensive and DOCA-salt-induced hypertensive rats over five weeks showed that saffron extract significantly reduced mean SBP in hypertensive rats in a dose-dependent manner but had no effect on normotensive rats. Additionally, the antihypertensive effects did not persist beyond the treatment period, suggesting continuous administration is necessary to maintain blood pressure-lowering benefits [41]. Additionally, another study showed that over five weeks, saffron (200 mg/kg/day) reduced the aorta's cross-sectional area, media thickness, and elastic lamellae number [42]. Recent studies indicated that crocin, crocetin, and safranal reduce oxidative stress by decreasing lipid peroxidation (malondialdehyde [MDA] levels) and nitric oxide (NO) levels and increasing glutathione, antioxidant enzymes (SOD, CAT, GPx), and thiol content [13]. This might be the mechanism for BP regulation.

Our analysis indicated no statistically significant difference in TNF levels between the intervention and control groups. However, there was considerable heterogeneity among the studies. In contrast with our findings, the study aimed to evaluate the effects of saffron aqueous extract (200 mg/kg body weight) on TNF-α levels in diabetic rats' liver, kidney showed that saffron aqueous extract normalized TNF-α levels in the liver and kidney tissues of diabetic rats, indicating its protective anti-inflammatory effect against hyperglycemia-induced inflammation [43]. Saffron and its active derivatives, such as crocin, crocetin, and safranal, exhibit significant immunoregulatory effects. Saffron has been shown to inhibit serum levels of key inflammatory markers, including nuclear transcription factor κB (NF-κB) p65 unit, TNF-α, interferon-gamma (IFN-γ), and interleukins (IL-1β, IL-6, IL-12, IL-17A). It functions as an antagonist of NF-κB and an agonist of peroxisome proliferator-activated receptor gamma (PPAR-γ). Furthermore, saffron down-regulates critical pro-inflammatory enzymes such as myeloperoxidase (MPO), cyclooxygenase-2 (COX-2), inducible nitric oxide synthase (iNOS), phospholipase A2, and prostanoids, thereby contributing to its overall anti-inflammatory and protective effects [44].

Laboratory liver tests such as AST and ALT are broadly defined as tests helpful in evaluating and treating patients with hepatic dysfunction [45]. Our findings indicated that saffron supplementation can significantly lower AST levels. Moreover, the heterogeneity among these studies was not significant. Conversely, the effect of saffron on ALT levels was less definitive and showed no statistically significant difference between the saffron and control groups. Furthermore, the analysis exhibited substantial heterogeneity, indicating high variability in the study outcomes. A study investigated the effects of 100 mg/day saffron supplementation on overweight/obese T2D patients in a double-blind, randomized trial over eight weeks. Results showed significant reductions in AST and ALT levels, indicating improved liver function [46]. Saffron (*Crocus sativus*) and its constituents, such as crocin, crocetin, and safranal, can protect the liver by activating the Nrf2/HO-1/Keap1 signaling pathway. This activation enhances anti-oxidant responses, reduces inflammation, and provides significant therapeutic effects [47]. Also, a study that investigated saffron's effects on liver inflammation and fibrosis using a CCl4-induced hepatic fibrosis mouse model showed 10 mg/kg and 20 mg/kg of saffron by inhibiting JAK/STAT3 phosphorylation in fibrotic livers, improved liver function, reduced inflammation, and decreased fibrosis [48].

Our study showed that saffron supplementation does not significantly affect creatinine or BUN levels, indicating a limited impact on renal function. Aligning with our research, the study by Norouzy et al. indicated that saffron supplementation had no significant effect on serum urea or serum creatinine levels compared to the placebo. Although there was a significant non-linear relationship with the duration of supplementation, saffron did not significantly affect renal function markers [49]. A study on kidney function in diabetic rats treated with 100 or 200 mg/kg of saffron extract daily for 28 days showed that saffron treatment significantly reduced urine volume and BUN at both doses but did not affect urinary total protein (UTP) and creatinine. Histological analysis showed improvements in renal damage, such as reduced thickening of the Bowman’s capsule membrane and less mesangial matrix expansion, suggesting saffron extract may help alleviate diabetic nephropathy through its antioxidant effects [50]. These differences in results may be due to variations in study design, dosage, duration, and the specific conditions of the subjects.

## Strengths and limitations

The present study has several important strengths. We have conducted a comprehensive assessment of saffron supplementation's effects on cardiometabolic risk factors in diabetic and prediabetic overweight patients. We included multiple RCTs from various populations, making the findings more generalizable. Moreover, we conducted several subgroup analyses to indicate the effects across different subgroups and to find out the potential sources of heterogeneity. Also, rigorous methodology, adhering to PRISMA guidelines and Jadad scale assessment, further ensured our study's reliability. Furthermore, by evaluating saffron supplementation's effects on various cardiometabolic indicators such as glycemic markers, lipid profile, BP, and inflammatory markers, the study offers a holistic view of saffron's potential health benefits. However, the study has some limitations. Several outcomes exhibited substantial heterogeneity, indicative of variations in study designs and populations, all of which may have impacted the consistency of the results. Additionally, excluding non-randomized studies and short follow-up periods may have led to the omission of relevant data. Publication bias in critical markers such as FBS, HbA1c, and HOMA suggests that the reported effects might be overestimated. Future research should reduce heterogeneity by standardizing intervention protocols and patient populations. Additionally, more RCT are needed to confirm the effects of saffron on various metabolic and inflammatory markers.

## Conclusion

Our systematic review and meta-analysis demonstrate that saffron supplementation significantly reduces fasting blood sugar (FBS), HbA1C, triglycerides (TG), systolic blood pressure (SBP), and aspartate aminotransferase (AST) in diabetic and prediabetic overweight individuals, highlighting its potential benefits in managing these cardiometabolic risk factors. However, saffron did not show significant effects on HDL, total cholesterol, LDL, insulin secretion, HOMA, diastolic blood pressure (DBP), TNF, alanine aminotransferase (ALT), creatinine, or blood urea nitrogen (BUN). Clinically, these findings suggest saffron could be an adjunctive therapy for improving glycemic control and certain aspects of cardiovascular health. Future research should focus on long-term studies, varied dosages, and different patient populations to further elucidate saffron's role and establish comprehensive guidelines for its clinical application.

## Supplementary Information

**Supplementary materials 1.**

## Data Availability

All data generated or analyzed during this study are included in this published article and its supplementary materials.

## Abbreviations

CMI: Cardiometabolic indicators
DM: Diabetes mellitus
T2DM: Type 2 diabetes mellitus
FBS: Fasting Blood Sugar
HbA1C: Hemoglobin A1C
HOMA-IR: Homeostasis Model Assessment
TG: Triglyceride
TC: Total Cholesterol
LDL: Low-density lipoproteins
HDL: High-Density Lipoprotein
SBP: Systolic blood pressure
DBP: Diastolic blood pressure
AST: Aspartate aminotransferase
ALT: Alanine aminotransferase
ALP: Alkaline phosphatase
Cr: Creatinine
BUN: Blood Urea Nitrogen
TNF-α: Tumor Necrosis Factor-alpha
