# Supplementary material for: Effects of saffron (Crocus sativus L.) supplementation on cardiometabolic Indices in diabetic and prediabetic overweight patients: a systematic review and meta-analysis of RCTs
Source: Diabetol Metab Syndr. 2024 Nov 27;16:286. doi: 10.1186/s13098-024-01530-6 (PMC11600967; doi:10.1186/s13098-024-01530-6)
Supplement: Supplementary file 1 — Supplementary materials 1. [file 13098_2024_1530_MOESM1_ESM.docx]

**Supplementary Materials**

**Query words:**

- **Saffron:** “Saffron” OR “Crocus” OR “Crocin” OR “Crocetin”
- **Cardiometabolic Indices:** “cardiometabolic” OR “Metabolic” OR “Metabolism” OR
- **Lipid profile:** “lipid profile” OR “blood lipid” OR “plasma lipid” OR “blood fat” OR “Lipemia*” OR “Lipidemia*” OR “Hyperlipemia” OR “Hyperlipidemia” OR “Hypolipemia” OR “Hypolipidemia” OR “Cholesterol” OR “Triglyceride*” OR “Triacylglycerol*” OR “lipoprotein” OR “lipoproteinemia” OR “HDL” OR “high-density lipoprotein” OR “LDL” OR “low-density lipoprotein” OR “Hypercholesteremia*” OR “Dyslipidemia” OR “Dyslipoproteinemia*” OR “hyperlipoproteinemia” OR “hyperlipidemic agents” OR “Apolipoprotein*” “alpha Lipoprotein” OR “Circulating Lipoprotein” OR “Lipid metabolism disorder”
- **Glycemic markers:** “glycemic” OR “blood glucose” OR “blood sugar” OR “plasma glucose” OR “glycemia” OR “Hyperglycemia” OR “Hypoglycemia” OR “FBG” OR “FBS” “Postprandial Hypoglycemia” OR “Insulin” OR “glucose tolerance test” OR “GTT” OR “OGTT” OR “HbA1C” OR “hemoglobin A1C” OR “glycohemoglobin*” OR “glycated hemoglobin test” OR “Glycosylated Hemoglobin A1c” OR “HOMA-IR” OR “HOMA-Index” OR “QUICKI” OR “Quantitative insulin sensitivity check index”
- **Inflammatory markers:** “inflammatory” OR “C-reactive Protein” OR “CRP” OR “High-sensitivity CRP” OR “hs-CRP” OR “Erythrocyte Sedimentation Rate” OR “ESR” OR “Interleukins” OR “ILs” OR “interleukin-6” OR “IL-6” OR “cytokines”
- **Oxidative stress:** “Oxidative” OR “Reactive Oxygen Species” OR “ROS” OR “Lipid Peroxidation Products” OR “Lipid Peroxidation Products” OR “DNA Damage Markers” OR “Antioxidant Enzymes” OR “superoxide dismutase” OR “SOD” OR “catalase” OR “CAT” OR “glutathione peroxidase” OR “GPx” OR “glutathione reductase” OR “GR” OR “Non-enzymatic Antioxidants” OR “Total Antioxidant Capacity” OR “TAC” OR “Advanced Oxidation Protein Products” OR “AOPPs” OR “F2-Isoprostanes” OR “prostaglandin”
- **Liver enzymes:** “liver test” OR “Alkaline phosphatase” OR “ALP” OR “Alanine transaminase” OR “ALT” OR “Aspartate transaminase” OR “AST” OR “Gamma-glutamyl transferase” OR “GGT”
- **Anthropometric:** “anthropometric” OR “Waist circumference” OR “Hip circumference” OR “weight” OR “hight”
- **Blood pressure**: “Blood pressure” OR “pulse pressure” OR “arterial blood pressure” OR “BP” OR “systolic pressure” OR “diastolic pressure” OR “hypertension” OR “hypotension”
- **Overweight**: “Obesity” OR “Obese” OR “Overweight” OR “Adiposity” OR “Weight gain” OR “Excess body fat” OR “Fat accumulation” OR “Body mass index”

**Table.** GRADE tool

| **Author/Year** | **Q1** | **Q2** | **Q3** | **Q4** | **Q5** |
| --- | --- | --- | --- | --- | --- |
| Tajaddini et al. 2023 | High | High | High | High | High |
| Kotanidouet al. 2023 | High | High | High | High | High |
| Sepahi et al. 2018 | High | Low | High | High | High |
| Rajabi et al. 2022 | Moderate | Low | High | High | High |
| Dastkhosh et al. 2022 | High | High | High | High | High |
| Jaafarinia et al. 2022 | High | High | High | High | High |
| Tajaddini et al. 2021 | High | High | High | High | High |
| Behrouz et al. 2020 | High | High | High | High | High |
| Mobasseri et al. 2020 | High | High | High | High | High |
| Karimi-Nazari et al. 2019 | High | High | High | High | High |
| Ebrahimi et al. 2019 | High | High | High | High | High |
| Aleali et al. 2019 | High | High | High | High | High |
| Sepahi et al. 2018 | High | Low | High | High | High |
| Milajerdi et al. 2017 | High | High | High | High | High |
| Azimi et al. 2014 | Moderate | low | High | High | High |

**Table.** Risk of Bias 2 (RoB-2)

| **Bias** | | | | | | |
| --- | --- | --- | --- | --- | --- | --- |
| **Author/Year** | **D1** | **D2** | **D3** | **D4** | **D5** | **Overall** |
| Tajaddini et al.  2023 | L | L | L | L | Some concerns | Some concerns |
| Kotanidouet al.  2023 | L | L | L | L | Some concerns | Some concerns |
| Sepahi et al.  2018 | L | L | Some concerns | L | Some concerns | Some concerns |
| Rajabi et al.  2022 | Some concerns | Some concerns | Some concerns | L | Some concerns | High |
| Dastkhosh et al.  2022 | L | L | Some concerns | L | Some concerns | Some concerns |
| Jaafarinia et al.  2022 | L | L | L | L | Some concerns | Some concerns |
| Tajaddini et al.  2021 | L | L | L | L | Some concerns | Some concerns |
| Behrouz et al.  2020 | L | L | L | L | Some concerns | Some concerns |
| Mobasseri et al.  2020 | L | L | L | L | Some concerns | Some concerns |
| Karimi-Nazari et al.  2019 | L | L | L | L | Some concerns | Some concerns |
| Ebrahimi et al  2019 | L | L | Some concerns | L | Some concerns | Some concerns |
| Aleali et al.  2019 | L | L | Some concerns | L | Some concerns | Some concerns |
| Sepahi et al.  2018 | L | L | Some concerns | L | Some concerns | Some concerns |
| Milajerdi et al.  2017 | L | L | L | L | Some concerns | Some concerns |
| Azimi et al.  2014 | L | L | L | Some concerns | Some concerns | Some concerns |

L: low-risk

**Publication bias**


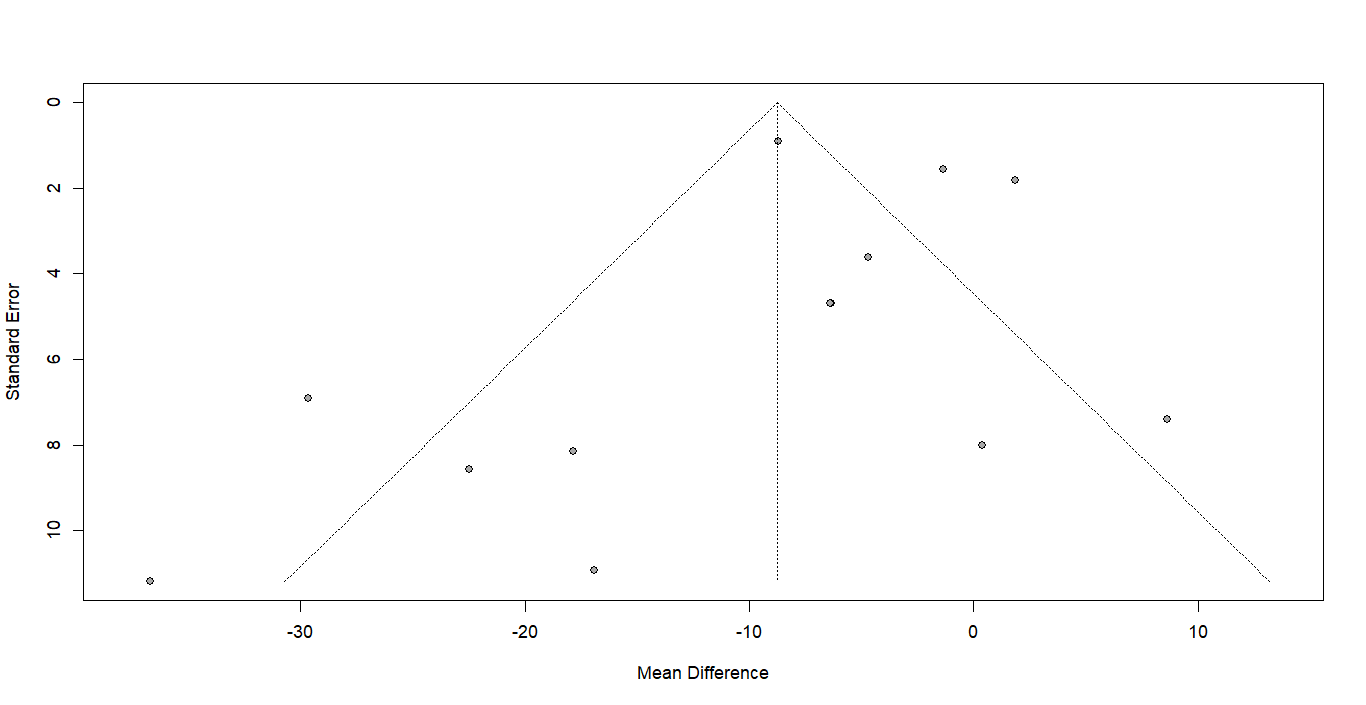


**FBS**


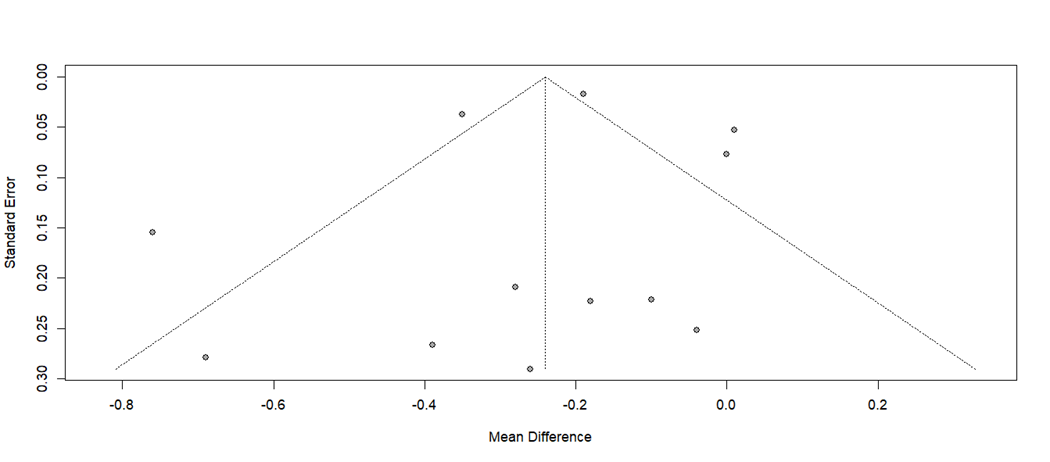


**HbA1C**


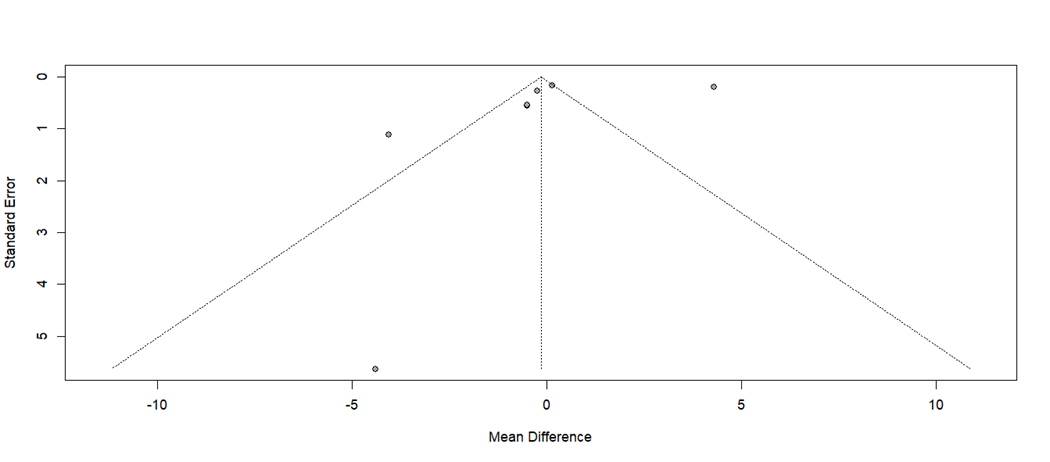


**Insulin**


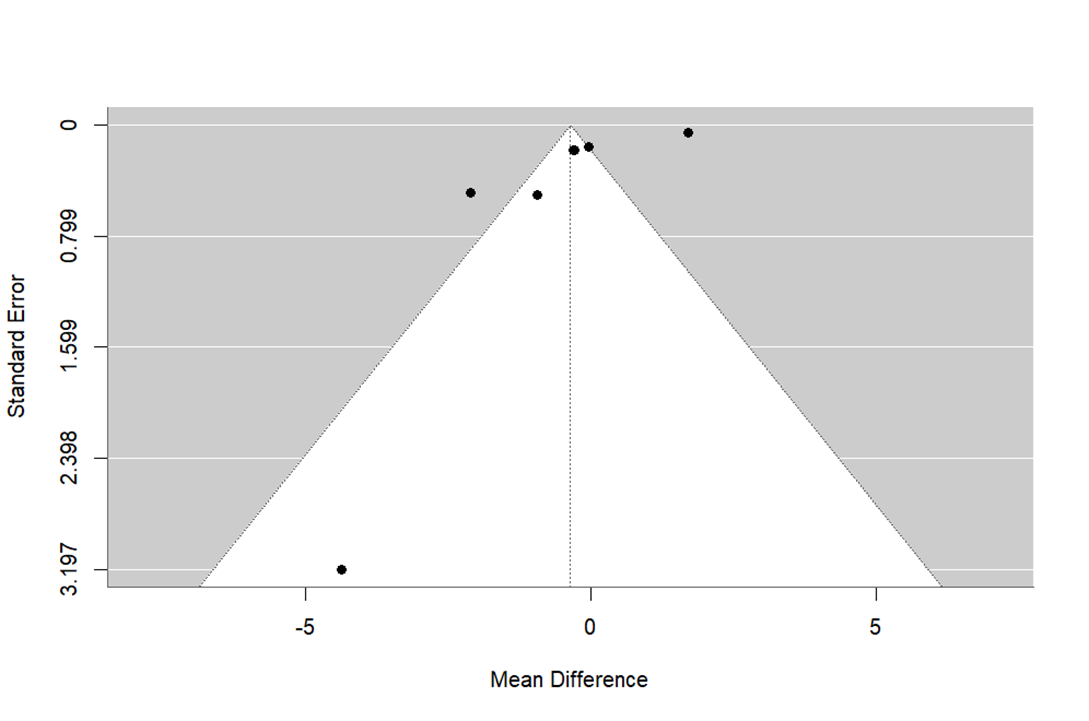


**HOMA**


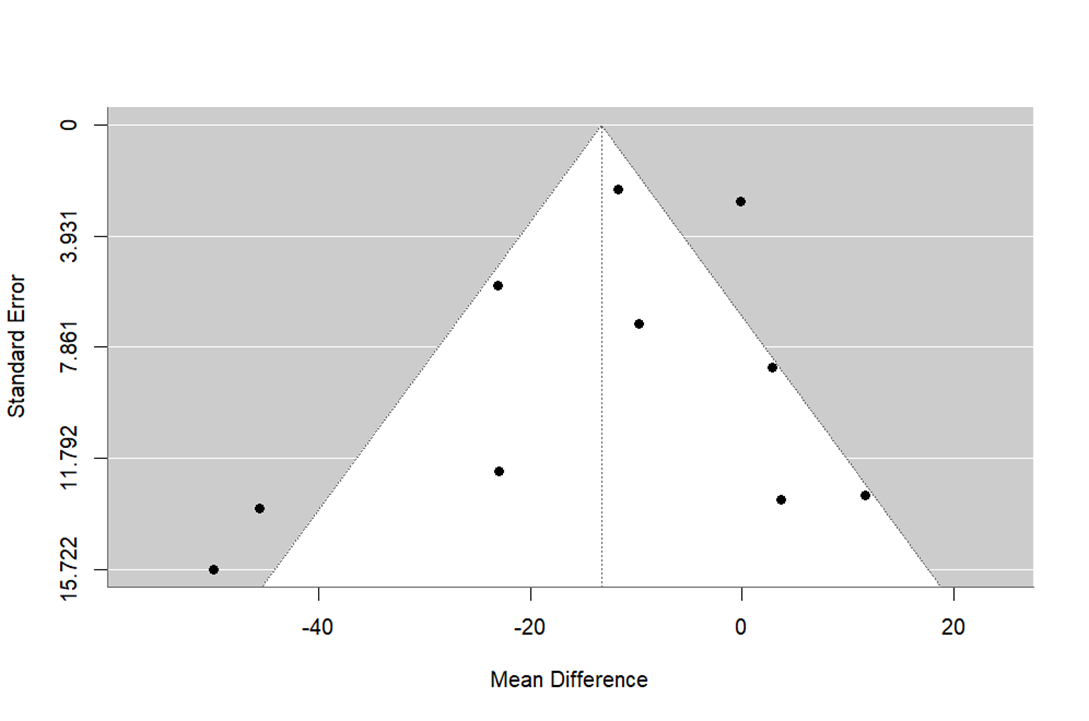


**Triglycerides (TG)**


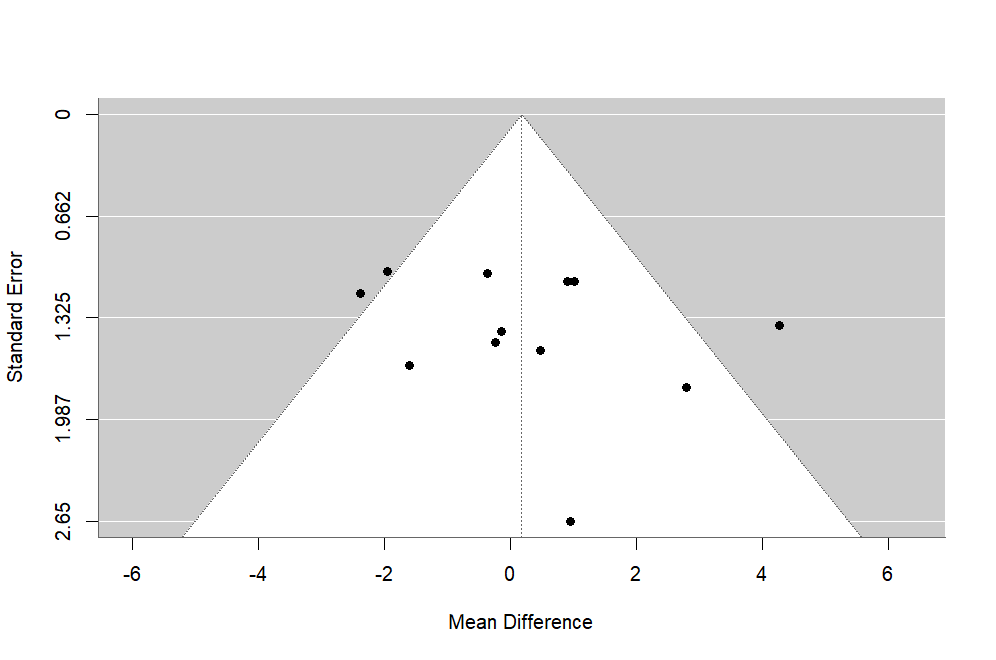


**HDL**


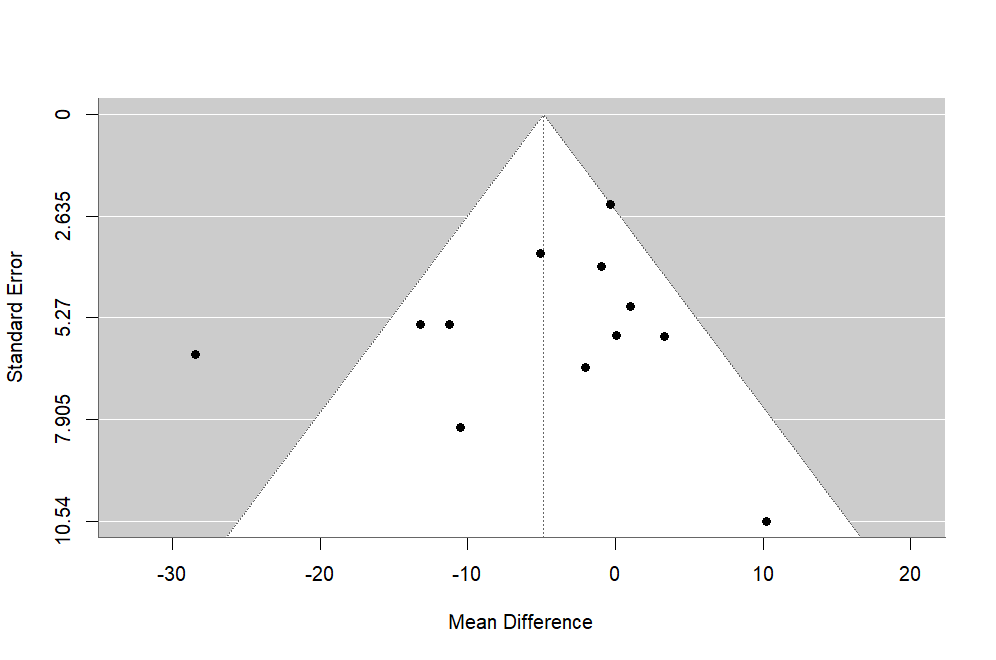


**Total Cholesterol**


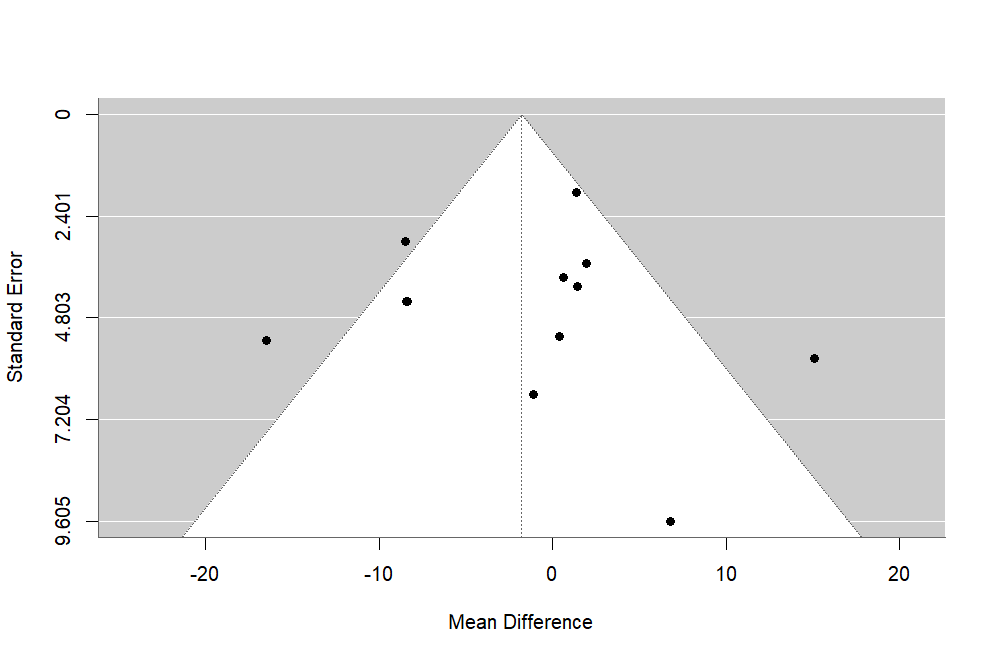


**LDL**


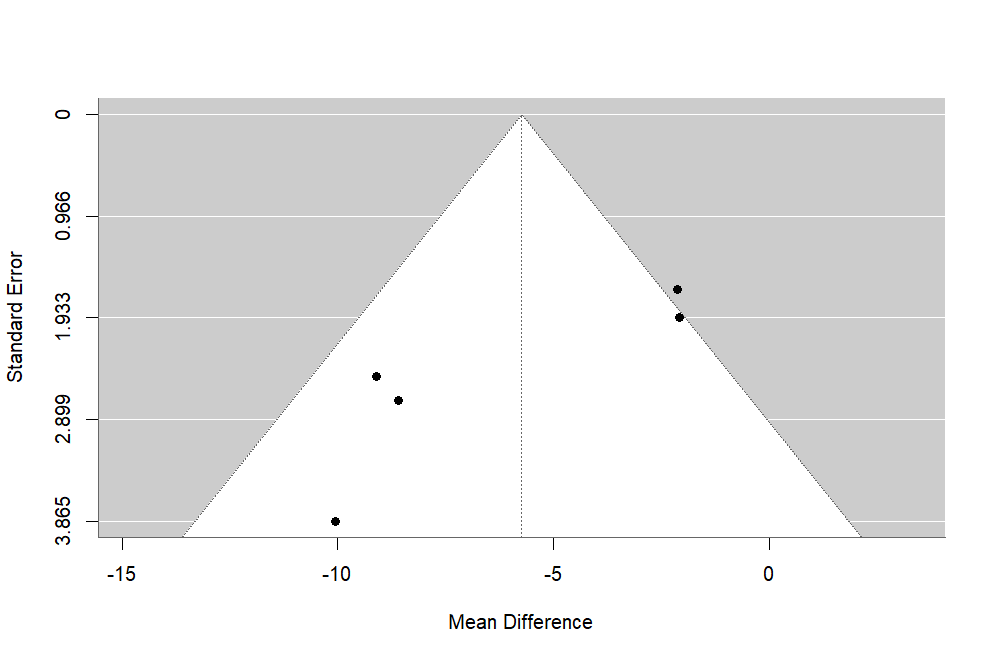


**Systolic blood pressure (SBP)**


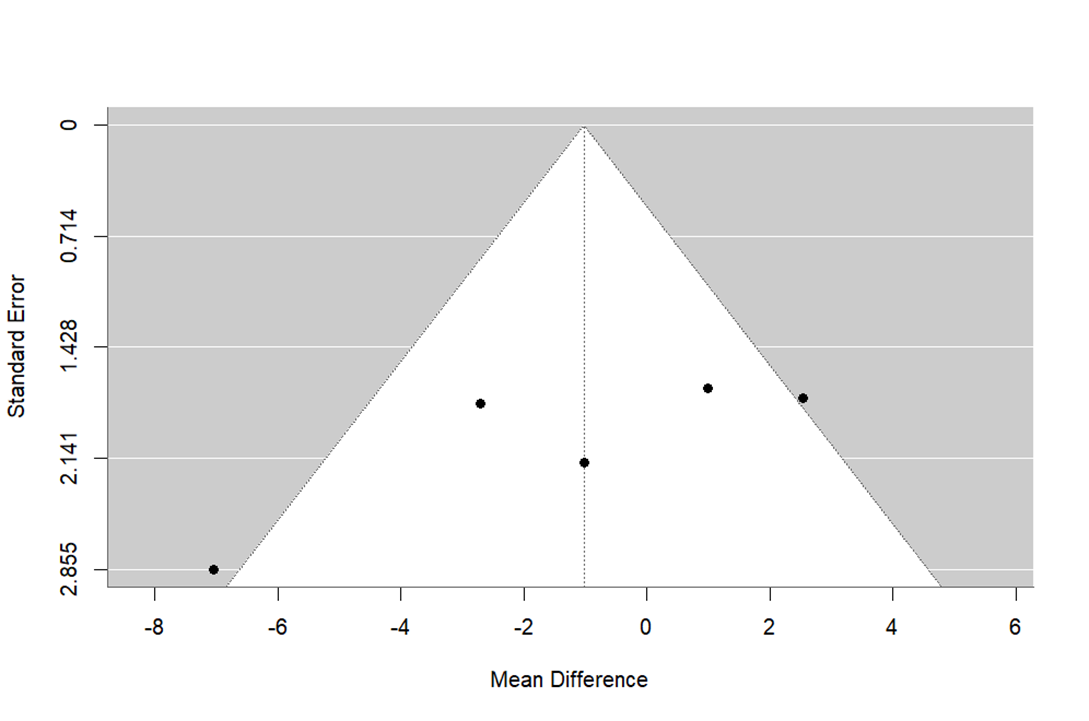


**Diastolic blood pressure (DBP)**


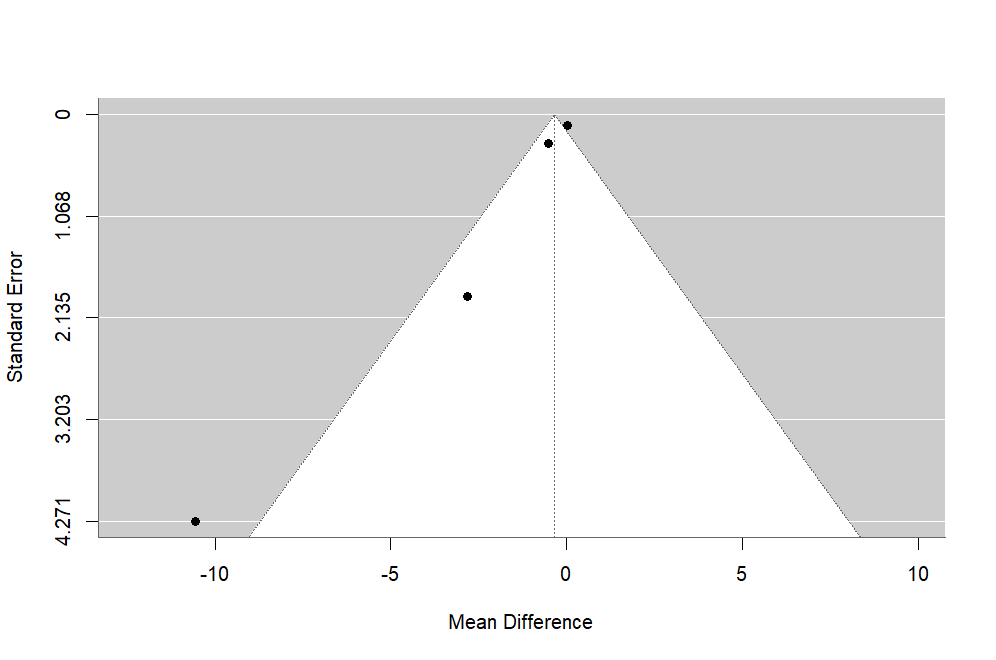


**TNF**


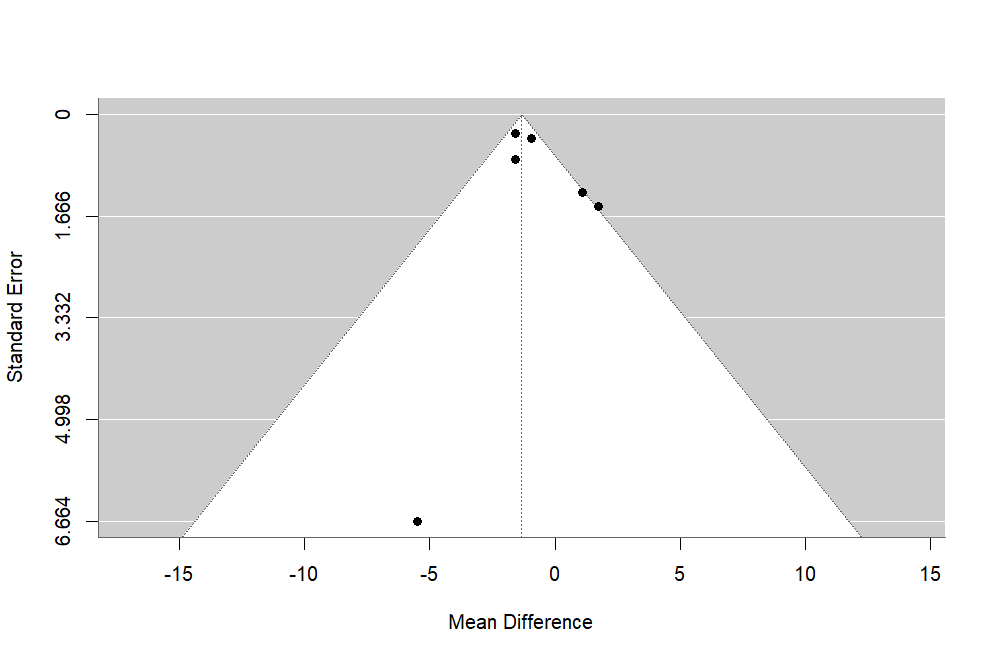


**AST**


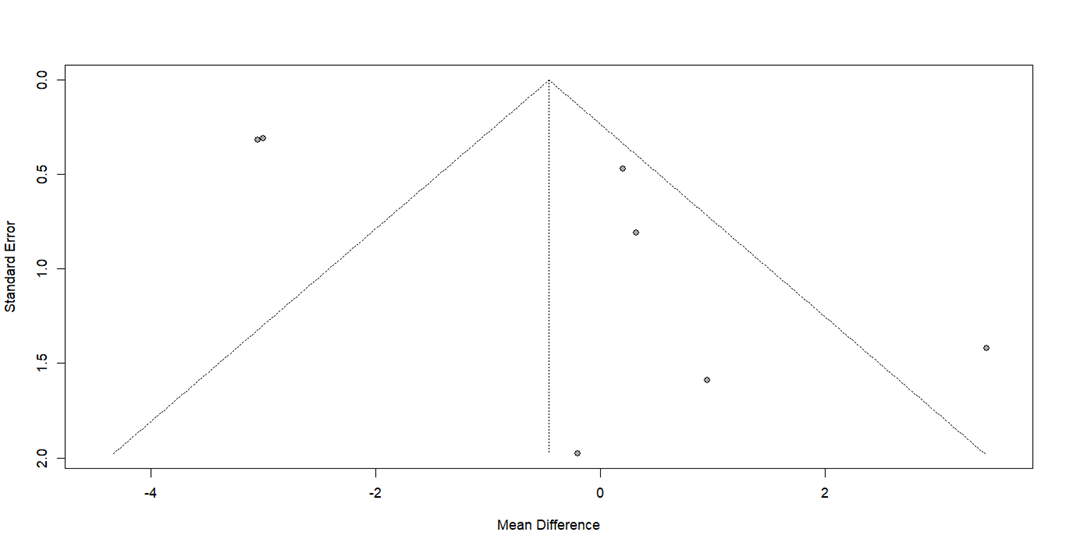


**ALT**


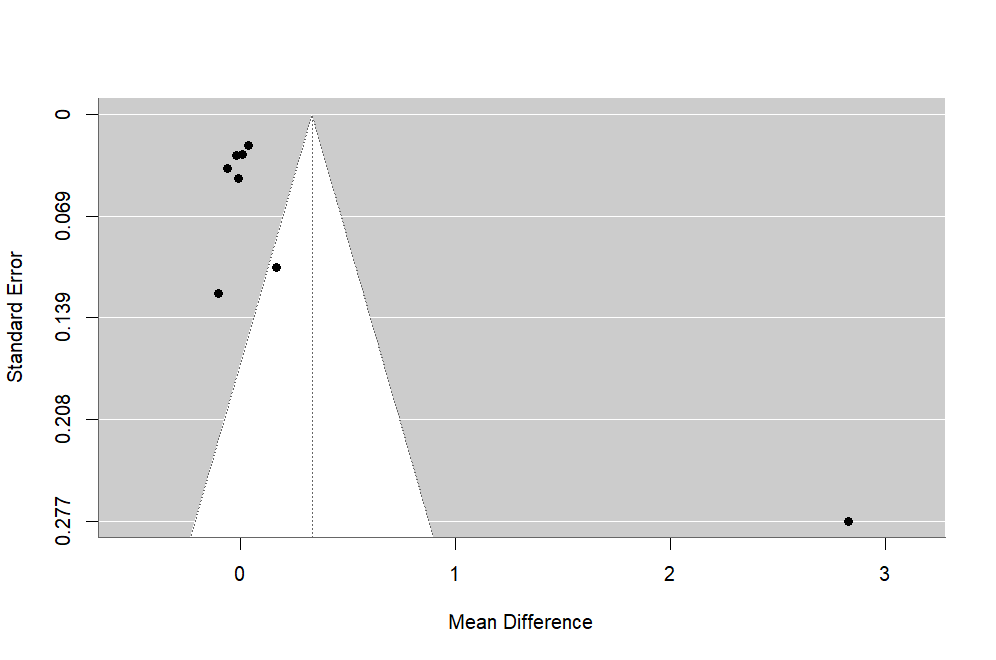


**CRP**


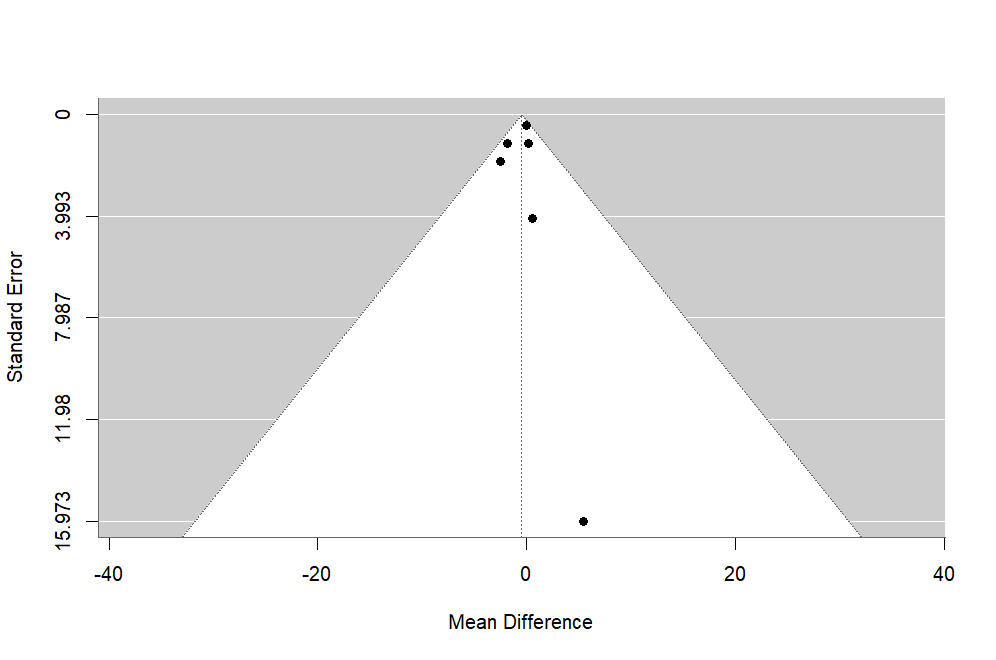


**BUN**
